# Effect of the Anchoring Layer and Transport Type on the Adsorption Kinetics of Lambda Carrageenan

**DOI:** 10.1021/acs.jpcb.1c03550

**Published:** 2021-07-13

**Authors:** Aneta Michna, Julia Maciejewska-Prończuk, Agata Pomorska, Monika Wasilewska, Tayfun Kilicer, Julia Witt, Ozlem Ozcan

**Affiliations:** † Jerzy Haber Institute of Catalysis and Surface Chemistry, Polish Academy of Sciences, Niezapominajek 8, PL-30239 Krakow, Poland; ‡ Bundesanstalt für Materialforschung und -prüfung, Unter den Eichen 87, 12163 Berlin, Germany

## Abstract

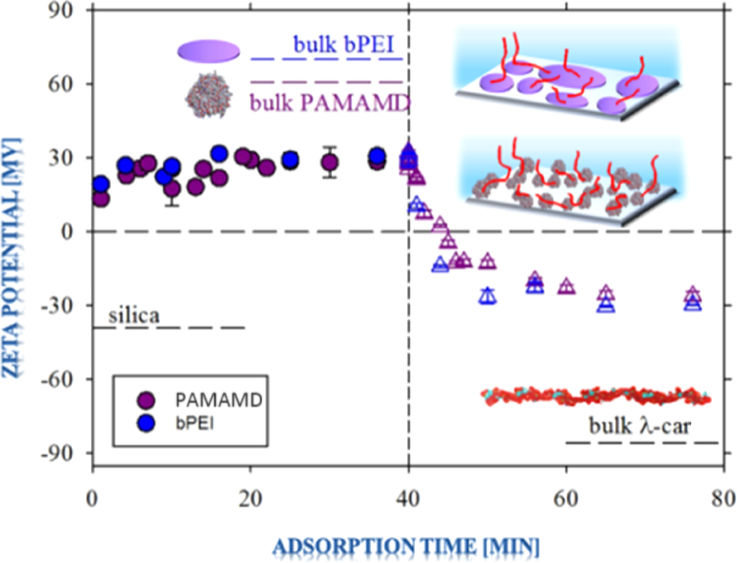

The kinetics of lambda carrageenan (λ-car) adsorption/desorption on/from anchoring layers under diffusion- and convection-controlled transport conditions were investigated. The eighth generation of poly(amidoamine) dendrimers and branched polyethyleneimine possessing different shapes and polydispersity indexes were used for anchoring layer formation. Dynamic light scattering, electrophoresis, streaming potential measurements, optical waveguide lightmode spectroscopy, and quartz crystal microbalance were applied to characterize the formation of mono- and bilayers. The unique combination of the employed techniques enabled detailed insights into the mechanism of the λ-car adsorption mainly controlled by electrostatic interactions. The results show that the macroion adsorption efficiency is strictly correlated with the value of the final zeta potentials of the anchoring layers, the transport type, and the initial bulk concentration of the macroions. The type of the macroion forming the anchoring layer had a minor impact on the kinetics of λ-car adsorption. Besides significance to basic science, the results presented in this paper can be used for the development of biocompatible and stable macroion multilayers of well-defined electrokinetic properties and structure.

## 1 Introduction

Carrageenans (Carrs) are an important class of natural polyelectrolytes of the polysaccharide family. On a commercial scale, they are mostly extracted from red seaweeds with water^1^ or hot alkali processes.^2,3^ Because of their nontoxicity, biocompatibility, and low cost Carrs are wildly used as thickeners, gelling agents, stabilizers, and emulsifiers in cosmetics and food products.^4−7^ In recent years, they have been increasingly used in pharmaceutical research for controlled drug release^8−10^ and in medicine as potent inhibitors of viruses such as herpes simplex virus,^11^ human immunodeficiency virus (HIV),^12^ and human papillomavirus (HPV).^13^ It was also demonstrated that Carrs reduce the duration of the common cold caused by other viruses.^14,15^ Furthermore, Carrs are currently investigated as potential therapeutic agents against SARS-CoV-2^16^ due to their antiviral properties.

Among Carrs, λ-carrageenan (λ-car) seems to be the most promising polysaccharide in terms of structure and solubility. It virtually has no anhydro-oxygen bridge residues, and therefore, it does not form a helix structure.^17^ λ-car contains three sulfate groups per disaccharide unit, making it the most negatively charged carrageenan. Furthermore, it does not form gels because it possesses no 3,6-anhydrogalactose residues.^7,18,19^ These properties lead to the high solubility of λ-car in water, even at low temperatures. Hence, λ-car can be employed as a thickener for the stabilization of food products such as synthetic milk, instant ice cream, or fruit drink.^19^ Moreover, this type of Carr can find application in drug delivery and release,^8,20,21^ showing antitumor and immunomodulation activities.^22^ It can serve as an agent preventing HPV infections,^13^ inhibiting HIV,^23^ and promoting apatite formation better than κ-carrageenan.^24^

The antibacterial properties of λ-car layers were reported by Briones *et al.*(25) The authors formed antibacterial coatings by sequential adsorption of polyethyleneimine (PEI) and λ-car. The multilayers were further analyzed by AFM, XPS, and biomolecular interaction analysis. It was found that the obtained coatings (successfully created *via* the LbL method), composed of 6 PEI/λ-car bilayers, were effective in inhibiting the growth of *Enterobacter cloacae*.^25^

It is worth underlining that the main advantage of the layer-by-layer (LbL) technique is the formation of the coatings of controlled composition and structure. Therefore, this technique was applied for the construction of λ-car-based macroion mono-, bi-, and multilayers in many studies.^26−31^ The multilayers were produced on various solid substrates such as mica,^25,26,28^ gold,^30,32^ silica,^27^ clay,^31^ or core template nanoparticles.^29^ The coatings were further analyzed using AFM,^25,27,28,30^ XPS,^25,26,30^ ellipsometry,^27,31^ polarimetry,^27^ circular dichroism,^27^ TEM,^29,31^ electrophoresis,^29^ contact angle measurements,^30^ and quartz crystal microbalance (QCM).^31,32^ These studies allowed the determination of the structure of adsorbed λ-car layers,^26−28^ the zeta potentials of nanocapsules containing λ-car,^29^ topography and roughness of the multilayers,^30^ or oxygen permeability of the multilayers.^31^

Despite the significance of λ-car and recent research efforts, the kinetics of the polysaccharide adsorption and desorption on a solid substrate, the effect of the anchoring layers on the formation of λ-car layers as well as electrokinetic properties of the λ-car layers formed under various mass transport conditions (diffusion and convection) remained unanswered. Bearing in mind the lack of adequate experimental data, the first goal of this research paper is to attain comprehensive physicochemical characteristics of λ-car as well as the synthetic macroions [branched PEI (bPEI) and the eighth generation of poly(amidoamine) dendrimers (PAMAMDs)] serving as the anchoring layers. These investigations were performed in electrolyte solution of defined ionic strength and pH using DLS and electrophoresis with laser Doppler velocimetry (ELDV). The detailed analysis of these macroions in bulk facilitated a quantitative interpretation of the anchoring layer properties *via* the determination of the λ-car adsorption/desorption kinetics and the electric properties of the bPEI/λ-car and PAMAMD/λ-car bilayers.

*In situ* streaming potential measurements (SPMs) and optical waveguide lightmode spectroscopy (OWLS) were applied for determining the dependence of the zeta potential of mono- and bilayers on macroion adsorption time, the kinetics of λ-car, bPEI and PAMAMD adsorption as well as the stability of their layers. The interpretation of the results obtained from these methods was supported by complementary studies by means of QCM with dissipation monitoring (QCM-D). AFM was applied to analyze the structure of the anchoring layers and the bilayers.

To the best of our knowledge, this is the first time that SPMs and OWLS are used for determining the physicochemical properties of bilayers containing λ-car and the effect of the anchoring layer and the transport type on the λ-car adsorption/desorption kinetics. We expect that the presented results can be exploited as a well-defined system, allowing for the quantitative interpretation of the adsorption/desorption kinetics of λ-car on the definite anchoring layer, which is of great importance for basic science and medical applications.

## 2 Experimental Methods

### 2.1 Materials

λ-car plant mucopolysaccharide with a molecular mass of 579,000 Da,^33^ branched bPEI (50% w/v aqueous solution) with a molecular mass of 70,000 Da, and the eighth generation of PAMAMDs (8.33% w/w aqueous solution, dispersity index below 1.04)^34^ with a molecular mass of 233,400 Da were purchased from Sigma-Aldrich (Poland), Polysciences Europe GmbH (Germany), and Dendritech, Inc. (the USA), respectively. The macroions were used as received. Ultrapure water (Milli-Q Elix & Simplicity 185 purification system, supplied by Millipore SAS Molsheim, France) and sodium chloride, NaCl, (analytical grade, pure p.a., Avantor Performance Materials Poland S.A) were used for the preparation of 0.01 M NaCl solution of pH 5.8.

The stock solutions of λ-car, bPEI, and PAMAMDs were prepared by dissolving the macroions in 0.01 M NaCl solution. The pH values of the obtained solutions were measured each time and adjusted with HCl (Sigma-Aldrich, Poland) to a pH value of 5.8. The low bulk macroion concentrations (1–5 mg L^–1^) were used for layer formation. To avoid the macroion depletion, caused by uncontrolled deposition onto glassware walls, the glass containing λ-car, bPEI, and PAMAMD solutions were conditioned three times for 15 min. The applied procedure allowed for the saturation of available adsorption sites present at the glass and to maintain constant bulk macroion concentrations. The experiments were carried at a temperature of 298 K.

The electrophoretic mobility and diffusion coefficient of the macroions were measured in folded capillary cells and disposable polystyrene cuvettes (Malvern Panalytical, United Kingdom), respectively. Silicon plates coated with silica, SiO_2_ wafers, (SIEGERT WAFER GmbH, Germany), glass sensors, OWLS sensors, (MicroVacuum Ltd., Hungary), silica-coated AT-cut quartz crystals, QCM sensors (QSense, Sweden) were applied as the substrates for the macroion adsorption experiments. The wafers and sensors were thoroughly cleaned directly before the experiments. The cleaning procedures are described in detail in our previous paper.^35^

AFM imaging was performed by using silicon cantilevers with a conductive Cr/Pt-coating (Multi75E-G-50: nominal spring constant of 3 N m^–1^, Budget Sensors, Canada, USA).

### 2.2 Methods

#### pH Measurements

The pH of NaCl and the macroion solutions were measured by a laboratory pH/conductivity/salinity meter (CPC-500, Elmetron, Poland).

#### DLS and Electrophoresis

The macroions were characterized in bulk by means of DLS and ELDV using a Malvern Zetasizer Nano ZS setup. These methods allowed the determination of macroion diffusion coefficients and electrophoretic mobilities in a constant ionic strength of 0.01 M NaCl at pH 5.8. The bulk concentrations of the macroions were equal to 100 mg L^–1^ (diffusion coefficient measurements) and 500 mg L^–1^ (electrophoretic mobility measurements). The bulk measurements were carried out at 298 K. The aforementioned parameters were applied for evaluating macroion hydrodynamic diameters and zeta potentials using the Stokes–Einstein^36^ and Henry equations,^37^ respectively.

#### Streaming Potential Measurements

The SPMs were performed in a parallel-plate channel formed by two SiO_2_ wafers separated by a Teflon spacer. The channel is the main part of the homemade cell equipped with a double Ag/AgCl electrode system. The streaming potential arises due to the forced hydrodynamic flow of the electrolyte through the channel. In the SPM measurements, the hydrodynamic flow was driven by the hydrodynamic pressure difference.

The streaming potential (Δ*E*_sp_) was measured *in situ* for four various hydrodynamic pressure differences (Δ*p*), which allows the monitoring of the dependence of Δ*E*_sp_ on Δ*p*. Using the Δ*E*_sp_*vs* Δ*p* slope, the zeta potentials of the bare SiO_2_ wafers and mono- and bilayer-covered SiO_2_ wafers were determined using the Smoluchowski equation,^38^ with the methodology reported in previous publications.^39−41^ The bulk concentrations of the macroion solutions were equal to 2 and 5 mg L^–1^, and the adsorption time was in the range of 1–40 min. The following procedure was applied to determine the apparent zeta potential of the macroion-covered SiO_2_ wafers:

(a) The streaming potential of SiO_2_ wafers was measured for the ionic strength of 0.01 M NaCl at pH 5.8.
(b) The macroion solution of PAMAMDs (or bPEI) was introduced into the cell channel and kept static for a selected duration (diffusion-controlled adsorption). In the case of the convection-controlled mass transport, the macroion solution was flowing through the channel for a set adsorption time at a constant volumetric flow rate (2.0 × 10^–2^ cm^3^ s^–1^). The macroion adsorption time was maintained in the range of 1–40 min for both transport types.
(c) The cell channel was washed with pure 0.01 M NaCl at pH 5.8.
(d) The streaming potential of silica covered either by the PAMAMD or bPEI layer was measured in 0.01 M NaCl at pH 5.8.
(e) λ-car solution was introduced into the channel. The λ-car molecules were adsorbed on the preadsorbed PAMAMD or bPEI layer under diffusion or convection-controlled mass transport conditions in the same way as described in (b). The λ-car adsorption time was in the range of 1–40 min for both mass transport types.
(f) The channel was flushed with pure 0.01 M NaCl at pH 5.8.
(g) The streaming potential of PAMAMD/λ-car- or the bPEI/λ-car bilayer-covered SiO_2_ wafers were measured in 0.01 M NaCl at pH 5.8.
(h) The measurements were repeated three times for all systems to check the reproducibility of the results. The differences among the experiment series were minor.
(i) The apparent zeta potentials of the bare SiO_2_ wafers as well as the macroion mono- (PAMAMD or bPEI) and bilayer (PAMAMD/λ-car or bPEI/λ-car)-covered SiO_2_ wafers were calculated using the Smoluchowski formula.^38^

The performed SPMs allowed for evaluating the kinetics of the adsorption/desorption of macroions.

#### OWLS Measurements

The label-free OWLS method allows the determination of the adsorbed “dry” mass of the macroions with sensitivity in the order of 1.0 × 10^–2^ mg m^–2^.^42,43^

The OWLS 210 apparatus (MicroVacuum Ltd., Hungary) operates with a laminar slit shear flow cell equipped with a planar waveguide (OW2400, MicroVacuum) formed by the glass (refractive index *n*_S_ = 1.52578) covered by a 170 nm coating of Si_0.78_Ti_0.22_O_2_ with a refractive index of *n*_F_ = 1.8. The macroion adsorption taking place on the waveguide surface is monitored *via* changes of the interfacial refractive index sensitive to the interfacial evanescent field of a He–Ne laser light coupled with diffractive grating on the waveguide surface.

Assuming that the layer is optically uniform, the adsorbed “dry” mass is calculated by using de Feijter’s formula^44^
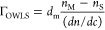
1where Γ_OWLS_ is the “dry” mass adsorbed on the sensor and is expressed in mg m^–2^, *d*_m_ is the thickness of the adsorbed layer, *n*_M_ is the refractive index of the layer, *n*_S_ is the refractive index of the solution measured by a refractometer, and *dn*/*dc* is the refractive index increment.

In this study, OWLS was employed for determining the effect of the anchoring layers on the adsorption/desorption kinetics of λ-car. The adsorption/desorption kinetics of PAMAMDs (or bPEI) on OWLS sensors were also analyzed.

A standard the *in situ* OWLS experiment involves three steps: establishing a baseline, adsorption, and rinsing (desorption) steps. Pure electrolyte (0.01 M NaCl) and macroion (PAMAMD, bPEI, and λ-car) solutions were introduced into the OWLS cell by a peristaltic pump. The solution flow rate was maintained constant at 2.5 × 10^–3^ cm^3^ s^–1^. After equilibration of the baseline, bilayer formation was monitored by pumping PAMAMD or bPEI solutions over the waveguide, followed by rinsing, pumping λ-car solution, followed by final rinsing. Rinsing between adsorption steps was started when adsorption equilibrium was attained. The ionic strength (0.01 M) and pH (5.8) were maintained constant in each adsorption step.

#### QCM-D Measurements

The kinetics of adsorption/desorption of PAMAMDs (or bPEI) on QCM sensors as well as λ-car on saturated PAMAMD (or bPEI) monolayer coated QCM sensors were studied using a Q-Sense E1 system (QSense, Gothenburg, Sweden) by following the standard procedure described in ref (45).

First, a stable baseline in the pure electrolyte (NaCl) at a constant ionic strength (same value for each phase of an experiment) was attained in the QCM-D cell for a defined electrolyte flow (1.33 × 10^–3^ cm^3^ s^–1^—same for the whole experiment time). Next, the solution of PAMAMDs or bPEI with a bulk concentration of 1 or 5 mg L^–1^ was introduced into the cell. The macroions from the solution flowing through the system with a defined velocity were adsorbed on the vibrating sensor surface. After 30 min of adsorption, the adsorbed layer was flushed with the pure electrolyte and the desorption process was monitored. Secondly, in the case of bilayer formation, 1 or 5 mg L^–1^ solution of λ-car was introduced into the cell. The adsorption of the second layer was carried out until a plateau was reached (around 100 min), followed by 30 min desorption during rinsing. The measurements were carried out at 298 K.

#### AFM Imaging

For AFM imaging, the mono- and bilayers were prepared *ex situ* under diffusion-controlled transport conditions. The procedure of the layer formation was as follows:

(a) SiO_2_ wafers were immersed in 5 mg L^–1^ of PAMAMD (or bPEI) solution of constant ionic strength of 0.01 M NaCl (pH 5.8) for 30 min and then thoroughly washed with pure 0.01 M NaCl (pH 5.8).
(b) Two freshly prepared SiO_2_ wafers covered by the PAMAMD (or bPEI) layer were placed in the cell to determine the zeta potential of macrocation-covered SiO_2_ wafers (modified wafers) in 0.01 M NaCl at pH 5.8.
(c) Directly after the SPMS, the modified wafers were rinsed with ultrapure water, dried in a stream of air, and imaged with AFM.
(d) To form macroion bilayers, modified wafers were submersed in 5 mg L^–1^ λ-car solution of 0.01 M NaCl (pH 5.8) for 30 min and then thoroughly washed with pure 0.01 M NaCl (pH 5.8).
(e) Two freshly prepared λ-car-covered modified wafers were placed in the cell to determine the zeta potential in 0.01 M NaCl at pH 5.8.
(f) After the measurements, the λ-car-covered modified wafers were rinsed with ultrapure water and dried in a stream of air.

Only one SiO_2_ wafer was placed in the 5 mg L^–1^ macroion solution for avoiding the depletion of the macroion. Each sample was prepared in fresh macroion solution.

The dry samples of modified wafers and λ-car-covered modified wafers were imaged under ambient conditions with NanoWizard 4 (JPK Instruments, Berlin, Germany) operating with a resolution of 512 × 512 pixels. The imaging was performed in intermittent mode at a scan frequency of 0.5–1.0 Hz using the Multi75E-G-50 (NanoAndMore GmbH, Germany) probe. JPK Data Processing Suite 6.1.88 was used for analysis of the topography images. Image flattening was performed with the second-order least-square polynomial function, which removes tilt and the vertical *z*-offset between line scans. The surface roughness values are given as the arithmetic average of the roughness profile (*R*_a_) in a total area of 5 μm × 5 μm.

## 3 Results and Discussion

The formation of macroion layers is mainly determined by electrostatic interactions. Thus, the thorough analysis of the bulk characteristics of λ-car, bPEI, and PAMAMD, including the size and zeta potential determination in defined ionic strength (0.01 M NaCl) and pH (5.8), is necessary to understand the kinetics of λ-car adsorption, the process of the macroion mono- and bilayer formation, and the structure of the adsorbed layers. The hydrodynamic diameter (hereafter referred to as size) and the zeta potential of λ-car and bPEI were determined from the measured diffusion coefficients and electrophoretic mobilities. The size and zeta potential of PAMAMD were previously determined for various pH values, ionic strengths, and electrolyte types as reported in our previous paper.^35^

For the convenience of the reader, the sizes, electrophoretic mobilities, and zeta potentials of the macroions are summarized in Table 1.

**Table 1:** Size (Hydrodynamic Diameter), Electrophoretic Mobility, and Zeta Potential of λ-car, bPEI, and PAMAMD Determined in 0.01 M NaCl at pH 5.8

macroion type	size [nm]	electrophoretic mobility [μm cm (V s)^−1^]	zeta potential [mV]	remarks
λ-car	69.7 ± 15.2	–4.49 ± 0.24	–85.8 ± 4.5	determined in this paper
bPEI	14.0 ± 3.8	3.66 ± 0.18	70.0 ± 3.5	determined in this paper
PAMAMD	10.2 ± 0.5	3.20 ± 0.40	60.8 ± 7.7	ref (35)

As can be seen in Table 1, the hydrodynamic diameters of λ-car and bPEI were equal to 69.7 nm ± 15.2 nm and 14 nm ± 3.8 nm, respectively. The λ-car hydrodynamic diameters agree with the size of ι-carrageenan reported by Thành *et al.,*(46) whereas the measured size of bPEI agrees well with the ones reported previously in the literature.^47−49^

It is worth underlying that the literature data also suggest that bPEI possesses a rather undefined shape making the size determination challenging.^49^

Contrary to bPEI, PAMAMDs possess a spherical shape in bulk.^50,51^ PAMAMDs showed a fairly narrow size distribution with an average size of 10.2 nm practically independent of the pH, ionic strength of the buffer, and simple electrolyte type.^35,52−54^

The electrophoretic mobilities and zeta potentials of bPEI and PAMAMD were 3.66 μm cm/V s and 70 mV and 3.20 μm cm/V s and 60.8 mV, respectively (see Table 1). These results confirm that in 0.01 M NaCl at pH 5.8, bPEI and PAMAMDs are strongly positively charged and, thus, should easily adsorb on negatively charged silica surfaces converting its surface charge to positive.

On the other hand, the measured electrophoretic mobility as well as the determined zeta potential of λ-car are strongly negative with values of −4.49 μm cm/V s and −85.8 mV, respectively. Considering electrostatic Columbic interactions, λ-car should easily adsorb on positively charged surfaces, that is, either the bPEI or PAMAMD layer. Typical λ-car, bPEI, and PAMAMD size distributions (derived from DLS), as well as the zeta potential distribution (derived from electrophoresis), are presented in Supporting Information (see Figure S1 and S2).

**Figure 1:**
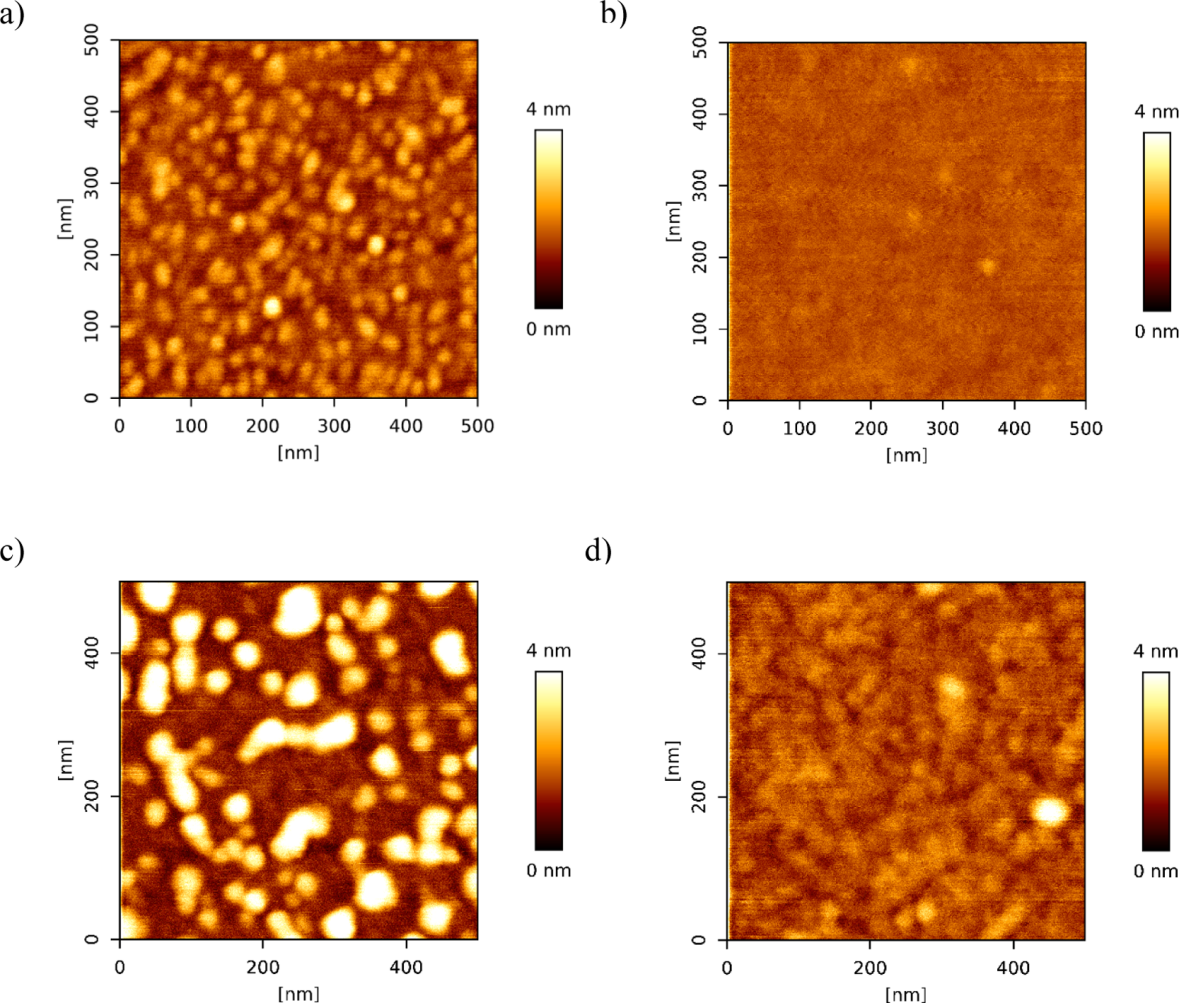
AFM images of the macroion monolayers (a) PAMAMD and (b) bPEI and bilayers (c) PAMAMD/λ-car and (d) bPEI/λ-car. The layers were adsorbed on silica under diffusion-controlled transport conditions: bulk macroion concentration 5 mg L^–1^, ionic strength 0.01 M, pH 5.8, and adsorption time 30 min. The scan size is 0.5 × 0.5 μm. The zeta potential of silica covered by macroion mono- (a,b) and bilayers (c,d) were equal to (a) 20, (b) 24, (c) −0.5, and (d) −13 mV.

The structures of PAMAMD and bPEI layers as well as PAMAMD/λ-car and bPEI/λ-car bilayers deposited on SiO_2_ wafers were evaluated using AFM imaging, as shown in Figure 1a–d. As indicated, PAMAMD and bPEI form layers with a completely different structure. The adsorbed PAMAMD molecules are well-separated, whereas bPEI molecules create a patchy structure (see Figure 1a,b). Bearing in mind the compression of the molecules by the atomic force microscope tip, one can postulate that PAMAMDs did not flatten significantly during adsorption on silica, whereas bPEI can notably flatten and form disks.

Our results are consistent with the ones reported by Pfau *et al.* who also suggested the high compression of bPEI during adsorption on mica.^55^ For bPEI with the molecular mass range of 37,000–150,000 Da, the heights of molecules were equal to 0.6 nm.^55^ It is worth noticing that Schneider *et al.* also reported the height of separated bPEI with a molecular mass of 750,000 Da adsorbed on mica between 0.5 and 1.5 nm.^56^ The authors also underlined the variation in sizes of adsorbed molecules due to polydispersity of bPEI in the solution. The high polydispersity of bPEI and the flattening of the molecules during adsorption on polyimide-coated glass were also reported by Saftics *et al.*(57)

Jackson *et al.*(54) investigated the radius of gyration of PAMAMDs (*R*_g_) by means of small-angle X-ray scattering by calculating a sphere radius (*R*) using the formula *R* = *R*_g_/√0.6. The PAMAMD sphere diameter (*d* = 2*R*) obtained with this methodology was further compared with the PAMAMD diameter (*d*_TEM_) determined by means of TEM. Both *d* and *d*_TEM_ were reported to be almost identical. Thus, one can postulate that PAMAMD only flattens slightly during adsorption. The degree of PAMAMD flattening depends on the adsorption time, as was shown in studies by Longtin *et al.*(58)

The zeta potentials of the PAMAMD and bPEI layers, presented in Figure 1a,b, were determined by the SPMs. The obtained values were similar, that is, 20 and 24 mV for PAMAMD and bPEI, respectively.

The AFM topography images presented in Figure 1c,d indicate that the structure of PAMAMD/λ-car and bPEI/λ-car bilayers varied significantly. Figure 1c shows the loosely packed structure of the PAMAMD/λ-car bilayer. The AFM results imply that the presence of “active centers”, containing a few PAMAMD molecules, situated very close to one another and possessing sufficiently high local electric charge is necessary for the efficient λ-car adsorption on PAMAMD-modified silica.

Oppositely to PAMAMD/λ-car, the bPEI/λ-car bilayer formed a highly ordered assembly (see Figure 1d), which is similar to the honeycomb structure formed by the λ-car layer on uncoated mica.^26^ Interestingly, the zeta potential of the PAMAMD/λ-car bilayer is equal to −0.5 mV, whereas that of the bPEI/λ-car bilayers is equal to −13 mV. The inversion of the charge from positive to negative confirmed that λ-car molecules were adsorbed on both PAMAMD and bPEI layers. The high polydispersity and shape (disk) of bPEI can have an impact on the high bPEI surface coverage.

The increase in the coverage of disks with polydispersity parameters was reported theoretically by Meakin and Jullien.^59^ They demonstrated that the jamming coverage of polydisperse disks increases proportionally to the power of 0.86 of the polydispersity parameter.^59^ On the other hand, the dimensionless jamming coverage of no interacting spheres characterized by a uniform size distribution in the bulk (like PAMAMDs) was constant.^60^

To gain a deeper understanding of the effects of the transport type and the anchoring layer on the kinetics of λ-car adsorption, SPMs were applied. As reported in previous publications, the SPMs provide information regarding the electrokinetic state of the macroion-covered macroscopic surfaces.^39−41,49^

The applied electrokinetic method allowed us to determine the zeta potential of the SiO_2_ wafer that was equal to −39 mV for the ionic strength of 0.01 M NaCl at pH 5.8. The obtained result agrees with the literature data, that is, −40^61^ and −35 mV.^41^ Knowing that PAMAMDs and bPEI are strongly positively charged and the SiO_2_ wafer as well as λ-car are negatively charged in 0.01 M NaCl at pH 5.8 (see Table 1), one can expect that alternating adsorption of PAMAMDs (or bPEI) and λ-car on the SiO_2_ wafer would lead to the deposition of PAMAMD (or bPEI)/λ-car bilayers.

The macroion adsorption was conducted under the diffusion- and convection (under the defined flow of the macroion solution)-controlled transport conditions.

If the adsorption is carried out under diffusion transport conditions, the surface concentration of the adsorbed solute (*N*) can be expressed using eq 2(62)
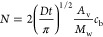
2where *D* is the diffusion coefficient, *t* is the solute adsorption time, *c*_b_ is the solute mass concentration in bulk (expressed as mg per L), *M*_w_ is the molar mass of the solute, and *A*_v_ is the Avogadro constant.

For convection-controlled transport conditions, *N* can be expressed as^62^
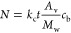
3where *k*_c_ represents the averaged mass transfer rate constant, and *k*_*c*_ = 1.165 ⟨*V*⟩ ^1/3^*D*^2/3^/*b*^1/3^*L*^1/3^ where ⟨*V*⟩ = *Q*/4*bc* is the solute mean flow rate through the streaming potential channel of dimensions 2*b* (width) × 2*c* (thickness) × *L* (length) and *Q* is the volumetric flow rate of the solute (expressed as cm^3^ per s).

As can be seen in eqs 2 and 3, *N* is directly proportional to *t*^1/2^ (for diffusion) and *t* (for convection).

It was assumed that globular PAMAMDs, disc-shaped bPEI, and λ-car do not undergo conformational changes after adsorption, and the variation of the zeta potential in time is attributed only to the changes of the adsorbed macroion amount. The macroions were irreversibly adsorbed on silica, as was stated in separate experiments (see Section 2 in Supporting Information)

The kinetics of adsorption can be assessed by plotting the change of the zeta potential as a function of the square root of the adsorption time if the macroion adsorption takes place under diffusion-controlled mass transport conditions. On the other hand, if the macroion adsorption is carried out under convective mass transport conditions, the zeta potential change is plotted against the adsorption time.

**Figure 2:**
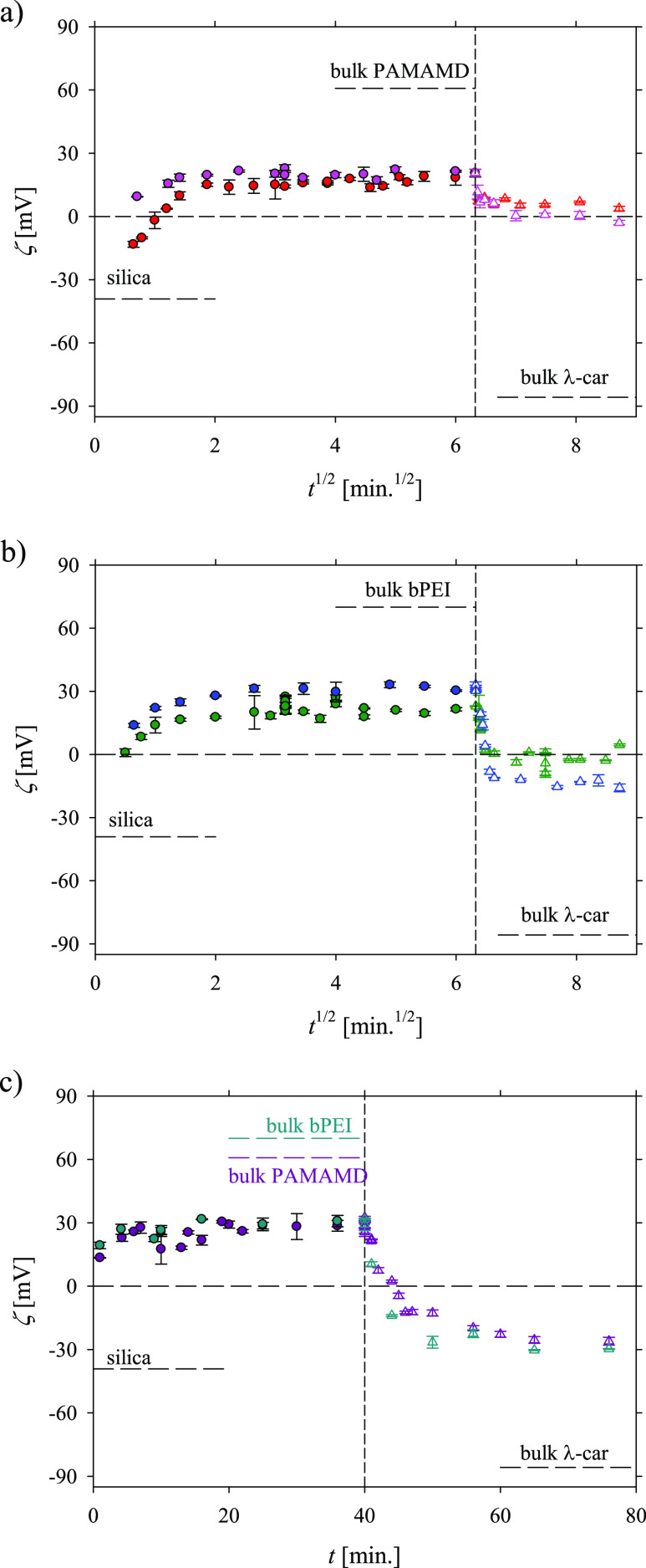
Kinetics of λ-car adsorption (red Δ, pink Δ, green Δ, dark blue Δ, violet Δ, light blue Δ) on the preadsorbed PAMAMDs (pink ●, red ●, violet ●) and bPEI layers (dark blue ●, green ●, light blue ●), respectively, presented as the change of the apparent zeta potential (ζ) as a function of the square root of adsorption time (*t*^1/2^) (a,b) and adsorption time (*t*) (c); *I* = 0.01 M NaCl, pH 5.8, and the initial bulk concentrations of the macroions (*c*_b_) were equal to 2 and 5 mg L^–1^, respectively. The applied symbols refer to the following systems: PAMAMD of *c*_b_ = 2 mg L^–1^, diffusion (red ●), PAMAMD of *c*_b_ = 5 mg L^–1^, diffusion (pink ●); λ-car of *c*_b_ = 2 mg L^–1^, diffusion (red Δ); λ-car of *c*_b_ = 5 mg L^–1^, diffusion (pink Δ), bPEI of *c*_b_ = 2 mg L^–1^, diffusion (green ●), bPEI of *c*_b_ = 5 mg L^–1^, diffusion (dark blue ●); λ-car of *c*_b_ = 2 mg L^–1^, diffusion (green Δ); λ-car of *c*_b_ = 5 mg L^–1^, diffusion (light blue Δ), PAMAMD of *c*_b_ = 5 mg L^–1^, convection (violet ●); bPEI of *c*_b_ = 5 mg L^–1^, convection (light blue ●); λ-car of *c*_b_ = 5 mg L^–1^, convection (violet Δ), λ-car of *c*_b_ = 5 mg L^–1^, convection (light blue Δ). (a) Formation of a complete PAMAMD/λ-car bilayer on silica, where after forming the first layer of PAMAMDs (*t*_1_ = 40 min), the second λ-car layer was deposited (*t*_2_ = 40 min). The experiment was carried out under diffusion-controlled transport conditions. (b) Formation of a complete bPEI/λ-car bilayer on silica, where after forming the first layer of bPEI (*t*_1_ = 40 min), the second λ-car layer was deposited (*t*_2_ = 40 min). The experiment was carried out under diffusion-controlled transport conditions. (c) Formation of complete PAMAMD/λ-car and bPEI/λ-car bilayers on silica, where after forming the saturated anchoring layer (PAMAMD or bPEI, *t*_1_ = 40 min), the λ-car layer was deposited (*t*_2_ = 40 min). The experiment was carried out under convection-controlled mass transport conditions. The volumetric flow rate of the macroions was constant and equal to *Q* = 2.0 × 10^–2^ cm^3^ s^–1^.

Typical results of the experiments carried out for PAMAMDs, bPEI, and λ-car of *c*_b_ equal to 2 and 5 mg L^–1^ in 0.01 M NaCl and pH 5.8, as shown in Figure 2a,b (diffusion) and Figure 2c (where the volumetric flow rate was equal to *Q* = 2.0 × 10^–2^ cm^3^ s^–1^).

As can be noticed, the formation of the PAMAMD and bPEI monolayers (full circles in Figure 2a,b) are related with a monotonic increase in the apparent zeta potential starting from the initial value of the bare SiO_2_ wafer to the maximum value of the apparent zeta potential of the SiO_2_ wafer covered by the suitable macrocation. The inversion in the sign occurs approx. 1 min after the macroion adsorption.

After 36 min of the adsorption, the average value of apparent zeta potentials of the PAMAMD- and bPEI-covered SiO_2_ wafer were 20 and 25 mV, respectively (see Figure 2a,b). The higher value of the apparent zeta potential of bPEI compared to PAMAMDs can be explained by the higher polydispersity of bPEI (allowing a higher bPEI surface coverage) and the larger diffusion coefficient of the disk shape (bPEI) that is 1.571 times larger than the sphere (PAMAMDs) shape of the same radius.^62^ The stability of the anchoring layers was also analyzed and is presented in Figure S3a,b in Supporting Information.

The adsorption kinetics of negatively charged λ-car on the anchoring layers are also presented in Figure 2a,b (to the right of the vertical dashed lines). As can be noticed, the apparent zeta potentials of PAMAMD/λ-car and bPEI/λ-car bilayers decreased exponentially with the square root of the adsorption time. The inversion of the substrate charge (from positive to negative) occurred after approx. 4 min of λ-car adsorption. The final values of the average apparent zeta potentials of the bilayers were 3 and −10 mV for PAMAMD and bPEI, respectively. Thus, one can conclude that the electrostatic interactions play a major role in macroion adsorption.

As shown in Figure 2c, the adsorption from both bPEI and PAMAMD solutions of the mass concentration of 5 mg L^–1^, under defined flow (*Q* = 2.0 × 10^–2^ cm^3^ s^–1^), did not significantly change, within the experimental error, the final value of the average apparent zeta potential 31 mV. It should be mentioned that similar trends were observed with a positively charged macrocation by Morga and Adamczyk.^63^ The authors reported that the mass transport type (*in situ* diffusion, *ex situ* diffusion, and *in situ* convection) has practically no impact on the final value of the apparent zeta potential of mica covered by the macrocation. The obtained final value of the zeta potential of bPEI-covered silica agrees with the one determined in studies by Mészáros *et al.*(64)

Also, a similar value of the apparent zeta potential of the PAMAMD-covered SiO_2_ wafer (25 mV) determined in 0.01 M at pH 5.5 was reported in our recent publication.^35^

Under convection-controlled mass transport conditions (see Figure 2c, to the right of the vertical dashed lines), the formation of both PAMAMD/λ-car and bPEI/λ-car bilayers results in an exponential decrease in the apparent zeta potential. The inversion of the substrate charge occurs 3 min after the introduction of λ-car solution into the cell. The final zeta potentials of the outer λ-car layers were very similar with values of −26 mV for PAMAMDs and −30 mV for bPEI. These values were lower than for the ones observed in experiments under diffusion-controlled mass transport with the same λ-car concentrations in the bulk. Thus, one can conclude that the process of λ-car layer formation is related not only to the value of the anchoring layer charge but also to the dominating mass transport type. It should be noted that fluid convection enhances particle transfer to the liquid–solid interface.^62^ During the convective flow, λ-car molecules are brought to the vicinity of the anchoring layers and λ-car concentrations are replenished (except for the diffusion boundary layer), which could explain the higher λ-car coverage.

The final zeta potentials of the λ-car films formed under the convective flow agree with the zeta potential of the nanocapsules with λ-car as the outer layer, which was reported as −31 mV.^29^ The λ-car layers were stable (see Supporting Information in Figure S3c,d).

The main conclusion that can be derived from the streaming potential results is that the convective mass transport, as well as the presence of an anchoring layer with a high positive surface charge, has a high impact on the adsorption kinetics of λ-car molecules and the final value of the zeta potential of the bilayers.

**Figure 3:**
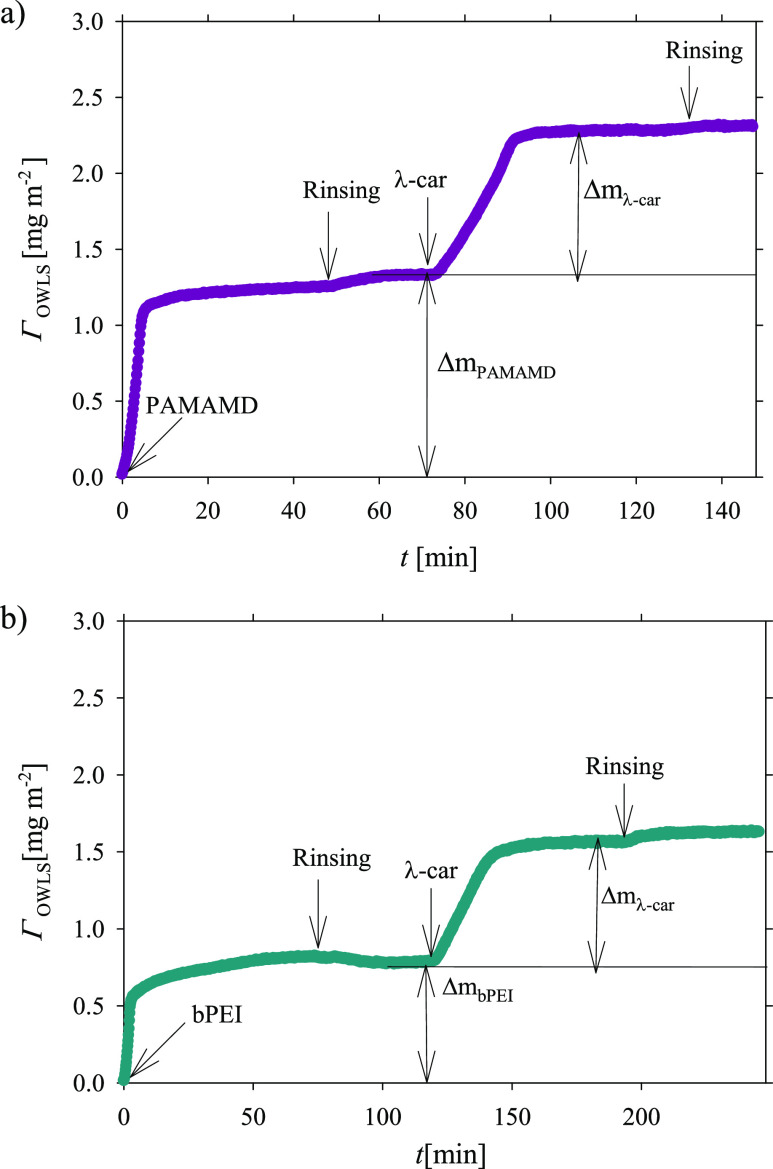
Adsorption kinetics of PAMAMD (a) (left side), bPEI (b) (left side), and λ-car on the preadsorbed (a) PAMAMDs (right side) and bPEI (b) (right side) layers. The dark pink and dark cyan lines represent the experimental data derived from the OWLS measurements for PAMAMDs and bPEI applied as the anchoring layers, respectively. The arrows indicate the beginning of the desorption runs. The mass concentration of PAMAMDs, bPEI, and λ-car was 5 mg L^–1^, ionic strength 0.01 M NaCl, pH 5.8, and volumetric flow rate 2.5 × 10^–3^ cm^3^ s^–1^.

Knowing the best conditions for the formation of highly packed anchoring monolayers and bilayers (*c*_b_ = 5 mg L^–1^, convection transport), the adsorption kinetics of λ-car and λ-car layer stability were analyzed by means of the OWLS technique. The series of the OWLS experiments followed the entire kinetics of adsorption/desorption of PAMAMDs, bPEI, and λ-car. The results shown in Figure 3 indicate the adsorption of PAMAMDs (Figure 3a) and bPEI (Figure 3b) at a linear rate (*t*) for short adsorption times, *t* < 5 min. The determined macroion monolayer masses (Γ_OWLS_) were equal to 1.0 and 0.57 mg m^–2^ for PAMAMD and bPEI, respectively. For *t* > 5 min, the increase in the adsorbed mass was slower until the limiting mass equal to 1.2 (PAMAMD) and 0.80 mg m^–2^ (bPEI) was reached. Then, the desorption run was initiated by rinsing the anchoring layer with a pure electrolyte (0.01 M NaCl, pH 5.8). Interestingly, after 30 min of washing, the PAMAMD mass slightly increased until 1.3 mg m^–2^, whereas the bPEI mass decreased to 0.7 mg m^–2^ (the irreversibly adsorbed fraction of bPEI).

PAMAMD mass uptake, observed at prolonged adsorption times, can be explained by the dendrimer conformational transition during prolonged adsorption.^58^ With prolonged adsorption times, the slightly deformed (adsorbed in the later stage of adsorption) and more deformed dendrimer (adsorbed in the initial stage of adsorption) fractions would be present on the surface. These different dendrimer conformations could have an impact on the measured *d*_m_ and *n*_M_ values and, thus, on the calculated Γ_OWLS_ (see eq 1).

On the other hand, the bPEI mass increase observed at prolonged adsorption times can be explained by the aggregation of the large bPEI molecules caused by the high polydispersity index of the macroions.^57^

It is worth noticing that for short adsorption times, the PAMAMD dendrimer mass (1 mg m^–2^) agrees with the results obtained by *in situ* reflectometry in studies by Cahill *et al.*(65) and Porus *et al.*(66) with reported PAMAMD adsorbed mass values of 1.2 and 1.1 mg m^–2^, respectively. These masses were acquired for the same adsorption conditions, that is, 5 min of adsorption, an initial bulk concentration of 5 mg L^–1^, pH 6.0, and the ionic strength of 0.01 M.

Reflectometry was applied for the determination of the bPEI adsorbed mass in studies by Mészáros *et al.*(64,67) For the ionic strength of 0.01 M, at pH 6.0 and *t* < 5 min, the adsorbed bPEI mass was equal to 0.4 mg m^–2^, which is 30% lower than the determined mass 0.56 mg m^–2^. These discrepancies can be explained by different solid substrates used for the bPEI adsorption: Si wafers with a 100 nm layer of SiO_2_ on the top (reflectometry) versus glass covered by a 170 nm layer of Si_0.78_Ti_0.22_O_2_ (OWLS). On the other hand, the mass determined for longer adsorption times (0.7 mg m^–2^) agrees with the mass of the bPEI saturated layer, that is, 0.1–0.7 mg m^–2^, determined from OWLS by Saftics *et al.*(57)

In the second step of the OWLS experiments, the adsorption/desorption kinetics of λ-car was studied on the preadsorbed stable PAMAMD and bPEI layers at the ionic strength of 0.01 M and pH 5.8. The results are the continuation of the kinetic traces shown in Figure 2a,b. The adsorption kinetics remained a linear function of time for λ-car adsorption times less than 20 min. After 20 min of adsorption, the stable adsorbed “dry” masses of the bilayers PAMAMD/λ-car and bPEI/λ-car were reached with values of 2.2 and 1.6 mg m^–2^, respectively. When the adsorption process was completed, the desorption run was initiated. The applied rinsing that lasted 20 min showed practically no changes in the determined λ-car deposited mass, indicating the high stability of the deposited films.

The irreversibly adsorbed (deposited) λ-car mass, referred to as “λ-car dry mass,” was calculated from the simple formula
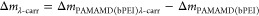
4where Δ*m*_λ-car_ is the adsorbed λ-car mass, Δ*m*_PAMAMD(bPEI)λ-car_ is the total mass of either the PAMAMD/λ-car (with PAMAMD as the anchoring layer) or bPEI/λ-car (with bPEI as the anchoring layer) bilayer, and Δ*m*_PAMAMD(bPEI)_ is the mass of the adsorbed anchoring layer (PAMAMD or bPEI).

The λ-car “dry” mass, determined using eq 4, was equal to 0.9 mg m^–2^ independent of the anchoring layer type. The OWLS results correlated very well with the streaming potential results obtained under convection-controlled mass transport conditions indicating that the adsorption of λ-car was irreversible during the experiment duration and confirmed that under convective mass transport conditions, the λ-car layer formation was independent of the anchoring layer type.

As mentioned earlier, the OWLS measurements allow for the determination of the “dry mass” of adsorbed layers. The QCM-D measurements deliver information about the “wet” mass containing both the “dry” mass and the mass of hydrodynamically coupled and internally associated solvent.^68^ The raw data of the QCM-D results that include the frequency changes (ΔFq) and dissipation shift (Δ*D*) as a function of time are presented in Supporting Information (see Figure S4). The basic QCM parameters are combined with the adsorbate mass uptake (a higher ΔFq indicates a higher adsorbed mass) and viscoelastic properties of the deposited layer (a higher Δ*D* indicates a softer and thicker adsorbed film).

**Figure 4:**
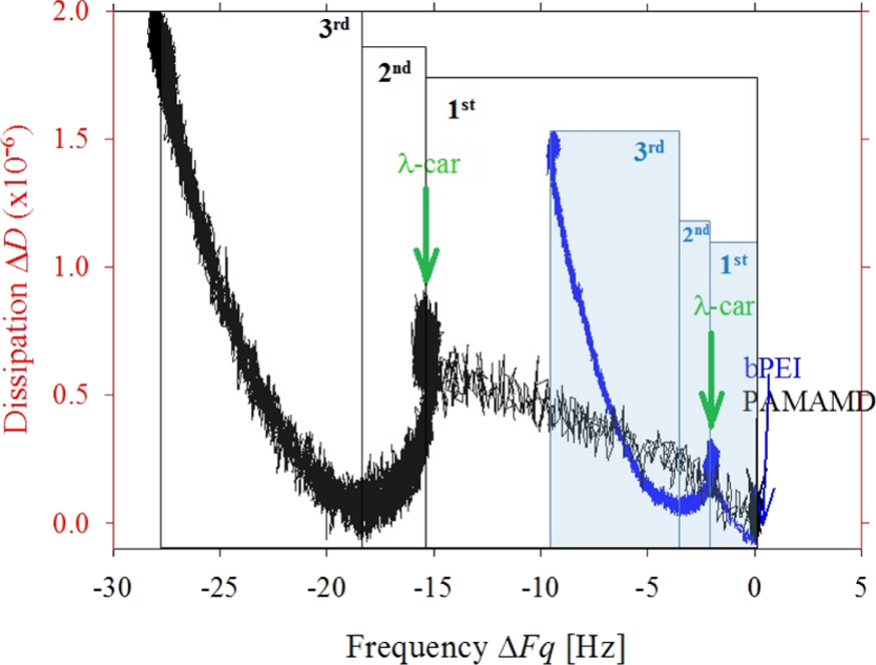
Dependence of the dissipation shift (Δ*D*) on frequency changes (ΔFq) determined for the formation of PAMAMD/λ-car (black curve) and bPEI/λ-car (blue curve) bilayers. The arrows show the start of the injection of PAMAMD (black), bPEI (blue), and λ-car solution (green). The bulk mass concentration of the macroions was equal to 1 mg L^–1^, ionic strength of 0.01 M, pH 5.8. The numbers 1st, 2nd, and 3rd indicate stages of the bilayer formation process. Based on the QCM-D results, the process of the PAMAMD/λ-car and bPEI/λ-car bilayer formation can be divided into three stages, as marked in the Fq–*D* plot (Figure 4).^69^

The QCM raw data are processed to an Fq–*D* diagram and presented in Figure 4 to emphasize structural deformations along bilayer formation at the quartz crystal surface as previously introduced in studies by Åkesson *et al.*(69) The dissipation shift is an interesting factor because it allows for interpreting the viscoelastic properties of the adsorbed layers. This parameter can be better analyzed when it is presented as a function of ΔFq (phase Fq–*D* diagram), along the adsorption of either the PAMAMD or bPEI layer, followed by the introduction of λ-car (Figure 4).

The dissipation increased almost linearly with the frequency decreasing during PAMAMD and bPEI adsorption in the 1st stage of the experiments (marked with number: 1st stage in Figure 4, dark-PAMAMD, blue-bPEI). The adsorption of the anchoring layer for 30 min led to the following ΔFq values for both macrocations: −15 Hz for PAMAMDs and −2 Hz for bPEI, whereas, the change in dissipation for both initial layers was lower than 1 × 10^–6^. The linear dependence of Δ*D* on ΔFq as well as the low value of Δ*D* (lower than 2 × 10^–6^) indicate that both types of the macrocations adsorb very fast on silica and form rigid monolayers.^70,71^

When λ-car was introduced (green arrows in Figure 4—2nd stage of bilayer formation), the frequency and the dissipation decreased to almost zero for both types of macrocations, implying tight adsorbate structure for both systems at this point. Further, the bilayer formation led to a decrease of ΔFq down to −28 Hz for PAMAMDs and −10 Hz for bPEI, in combination with a substantial Δ*D* increase up to 2 × 10^–6^ for PAMAMDs and 1.5 × 10^–6^ for bPEI in the 3rd stage. It was evident that λ-car adsorption on the PAMAMD layer led to a heavier (lower ΔFq values, dark curve in Figure 4) and viscous/soft (higher Δ*D* value) bilayer than the one formed on the bPEI layer (blue curve, Figure 4). However, it should be noted that the Δ*D* value was still in the low dissipation regime and both bilayer types are rather rigid.^70,71^

Because the dissipation shift (Δ*D*) for these systems was within the applicability range of the Sauerbrey equation,^70^ the “wet mass” of the macroions (Γ_QCM_) was calculated using the Sauerbrey formula.^72^

**Figure 5:**
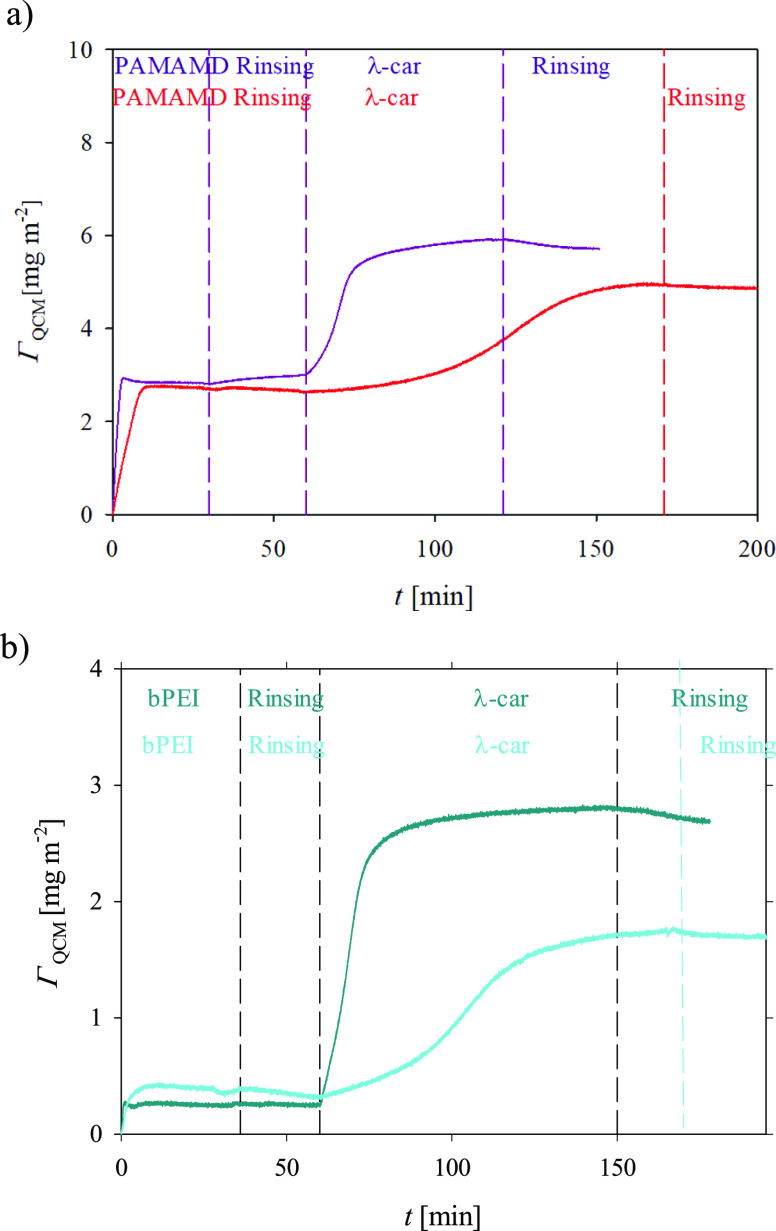
Kinetics of the λ-car adsorption on (a) PAMAMD- and (b) bPEI-covered silica sensors derived from QCM-D measurements using the Sauerbrey equation. The mass concentration of the macroions was equal to 1 mg L^–1^ (red and cyan curves) and 5 mg L^–1^ (dark pink and dark cyan), ionic strength of 0.01 M, pH 5.8, and volumetric flow rate 1.33 × 10^–3^ cm^3^ s^–1^.

Figure 5 shows the entire adsorption/desorption runs (Γ_QCM_ – wet mass in mg m^–2^ as a function of time calculated from the same raw data as the diagram in Figure 4) of PAMAMD/λ-car and bPEI/λ-car bilayers determined for the bulk concentrations of the macroions of 1 and 5 mg L^–1^ acquired by QCM-D.

As shown in Figure 5, the saturated “wet” masses formed under convection-controlled mass transport conditions for bPEI and PAMAMDs for long adsorption times are practically independent of the initial bulk mass concentration of the macroions (1 and 5 mg L^–1^). They were equal to 2.8 (see Figure 5a) and 0.3 mg m^–2^ (see Figure 5b) for PAMAMDs and bPEI, respectively. The obtained wet mass of saturated PAMAMD layers agrees with the one determined in our previous publication for the same dendrimer type of a bulk concentration of 1 mg L^–1^ at pH 5.8 and for the ionic strength of 0.01 M.^35^ It is worth mentioning that the same adsorbed mass of bPEI with a molecular mass of 70 kDa, evaluated at pH 6.0 for the initial bulk concentration of 500 mg L^–1^, was obtained in studies by Elzbieciak *et al.*(73)

Despite the same adsorbed wet masses of saturated PAMAMD and bPEI layers, the maximum masses of the bilayers, obtained from various bulk concentrations, differed significantly. As shown in Figure 5a, if λ-car was adsorbed from the PAMAMDs of a bulk concentration of 1 mg L^–1^, the maximum mass of PAMAMD/λ-car was equal to 4.9 mg m^–2^ (red curve), whereas due to adsorption from 5 mg L^–1^, the bilayer mass increased to 5.7 mg m^–2^ (dark pink). The bilayers formed on bPEI layers, having almost identical masses, showed similar behavior. The maximum saturated mass of bPEI/λ-car increased from 1.7 (cyan curve) to 2.7 mg m^–2^ (dark cyan curve) when the λ-car bulk concentration increased from 1 to 5 mg L^–1^.

The increase in the adsorbed mass with the initial bulk concentration of poly-l-lysine, having an elongated shape,^74^ was also observed in studies by Kosior *et al.*(75) Similar to the results obtained from the OWLS, no desorption of λ-car molecules from both bilayer types was observed.

Quantitatively, the water content (*H*) of the layers can be determined using the “wet mass” (from QCM-D) and the “dry mass” (from OWLS) by introducing a simple relationship defined as
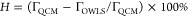
5

The water factors were equal to 57 and 53% for the PAMAMD layer and PAMAMD/λ-car bilayer, respectively. These results indicate that highly hydrated mono- and bilayers were formed on the silica surfaces in agreement with the literature data where total hydration of PAMAMD layers in the range of 50–80% was reported.^66,76,77^

The determination of the water factors for bPEI and bPEI/λ-car layers was not performed due to the possible aggregation of the large bPEI molecules (note the high polydispersity of bPEI) occurring due to long adsorption in OWLS experiments. Accordingly, the “dry” mass (0.7 mg m^–2^) of adsorbed bPEI determined from the long-time OWLS experiments was higher than the bPEI “wet” mass (0.3 mg m^–2^) evaluated in a shorter period of time (30 min) during QCM-D measurements. However, according to the literature, the water content of the bPEI layer is expected to be much lower (*H* = 6%).^78^

As shown in Figure 5, there are significant differences between the wet mass of the adsorbed PAMAMD and bPEI monolayers and of their bilayers with λ-car. These differences can be explained by the high hydration of the PAMAMD layer (*H* = 57%) and PAMAMD/λ-car bilayers (*H* = 53%) and the low hydration of the bPEI layer (*H* = 6%). Furthermore, one can postulate that λ-car chains, depending on the initial bulk concentration of λ-car, can adsorb in different confirmations. For the low initial bulk concentration of λ-car, the chains can adsorb only in the “side-on” conformation. The lower macroion coverage (lower “wet mass”) is attained due to the high repulsion between the adsorbed chains. For the higher initial λ-car bulk concentration, the chains can reorganize on the surface and change their conformation. The “end-on” conformation of the chains would then be the preferred mode of adsorption. When the λ-car chains adsorbed in the “end-on” conformation, they formed highly hydrated “quasi-polymeric brushes”. However, further research is required to confirm the abovementioned hypothesis.

It is worth underlining that the experimental data obtained from the SPMs and QCM-D can be interpreted in terms of the theoretical results obtained by coupling the random sequential adsorption blocking function with the bulk transport equation. It allows for formulating a general mass transfer equation whose solutions describe the theoretical dependencies of the solute coverage (Γ) on the adsorption time. This model was discussed in detail in a book chapter.^62^

## 4 Conclusions

The physicochemical parameters of λ-car, PAMAMDs, and bPEI, including the hydrodynamic diameters and zeta potentials, were investigated under conditions of defined ionic strength and pH.

For the first time, both the kinetics of λ-car adsorption on macroion anchoring layers and the stability of the formed bilayers were evaluated. Adsorption kinetics was analyzed under both diffusion and convection-controlled mass transport conditions by SPMs, OWLS, and QCM. The morphology of the formed bilayers was investigated by means of AFM.

The results demonstrate that λ-car can form both loosely and highly packed structures depending on the anchoring layer type. The streaming potential studies revealed that λ-car effectively adsorbs on the highly positively charged surface. Under convection-dominated mass transport conditions, the type of the macroion forming the anchoring layer had practically no impact on the kinetics of λ-car adsorption and the final zeta potential of the bilayer. These results are well correlated with the identical maximum λ-car “dry” mass measured for both anchoring layer types under convection-controlled mass transport conditions by means of OWLS.

The plateau values of maximum “wet” adsorbed masses of the macroions were determined using QCM-D. The maximum “wet” masses of PAMAMDs, bPEI, PAMAMD/λ-car, and bPEI/λ-car layers were dependent on the macroion type, which can be explained by the high hydration (reaching 60%) of PAMAMDs, PAMAMD/λ-car, and bPEI/λ-car layers and the low hydration of the bPEI layer (*H* = 6%). Significant differences between the wet mass of the bilayers with λ-car observed with different initial bulk concentrations of λ-car can be explained by conformational changes of the formed λ-car layer. For low initial bulk concentrations of λ-car, the chains tend to adsorb in the “side-on” conformation, whereas for high bulk concentrations—“end-on” conformation is preferred and the adsorbed λ-car chains formed highly hydrated quasi “polymeric brushes”.

Moreover, evaluating the QCM time-dependent data in the form of an Fq–*D* diagram allowed us to confirm that λ-car adsorption on the PAMAMD layer leads to a heavier and more viscous/soft bilayer than the one built on the bPEI layer.

Finally, it can be concluded that the λ-car adsorption on a solid substrate is irreversible and is mainly controlled by the electrostatic interactions, transport type, and initial bulk concentration of λ-car.

Besides significance to basic science, the data obtained in this paper can be used for developing biocompatible and stable macroion multilayers of well-defined electrokinetic properties and structure.

## Supporting Information Available

Size and zeta potential distributions of the macroions; stability of the macroion layers determined from SPMs; and frequency (ΔFq) and dissipation (Δ*D*) shift as a function of macroion adsorption time (PDF)

## References

[ref1] Montolalu R. I.; Tashiro Y.; Matsukawa S.; Ogawa H. Effects of Extraction Parameters on Gel Properties of Carrageenan from Kappaphycus Alvarezii (Rhodophyta). J. Appl. Phycol. 2008, 20, 521–526. 10.1007/s10811-007-9284-2.

[ref2] Encyclopedia of Food Sciences and Nutrition; Caballero B., Trugo L. C., Finglas P., Eds.; Academic Press, 2003.

[ref3] Bono A.; Anisuzzaman S. M.; Ding O. W. Effect of Process Conditions on the Gel Viscosity and Gel Strength of Semi-Refined Carrageenan (SRC) Produced from Seaweed (Kappaphycus Alvarezii). J. King Saud Univ.-Eng. Sci. 2014, 26, 3–9. 10.1016/j.jksues.2012.06.001.

[ref4] Karim A.; Sulebele G. A.; Azhar M. E.; Ping C. Y. Effect of Carrageenan on Yield and Properties of Tofu. Food Chem. 1999, 66, 159–165. 10.1016/s0308-8146(98)00258-1.

[ref5] Saha D.; Bhattacharya S. Hydrocolloids as Thickening and Gelling Agents in Food: A Critical Review. J. Food Sci. Technol. 2010, 47, 587–597. 10.1007/s13197-010-0162-6. 23572691 PMC3551143

[ref6] Dickinson E.; Pawlowsky K. Effect of ι -Carrageenan on Flocculation, Creaming, and Rheology of a Protein-Stabilized Emulsion. J. Agric. Food Chem. 1997, 45, 3799–3806. 10.1021/jf970304d.

[ref7] Bhardwaj T. R.; Kanwar M.; Lal R.; Gupta A. Natural Gums and Modified Natural Gums as Sustained-Release Carriers. Drug Dev. Ind. Pharm. 2000, 26, 1025–1038. 10.1081/ddc-100100266. 11028217

[ref8] Hariharan M.; Wheatley T. A.; Price J. C. Controlled-Release Tablet Matrices from Carrageenans: Compression and Dissolution Studies. Pharm. Dev. Technol. 1997, 2, 383–393. 10.3109/10837459709022637. 9552467

[ref9] Picker K. M. Matrix Tablets of Carrageenans II. Release Behavior and Effect of Added Cations. Drug Dev. Ind. Pharm. 1999, 25, 339–346. 10.1081/ddc-100102179. 10071827

[ref10] Li L.; Ni R.; Shao Y.; Mao S. Carrageenan and Its Applications in Drug Delivery. Carbohydr. Polym. 2014, 103, 1–11. 10.1016/j.carbpol.2013.12.008. 24528694

[ref11] González M. E.; Alarcón B.; Carrasco L. Polysaccharides as Antiviral Agents: Antiviral Activity of Carrageenan. Antimicrob. Agents Chemother. 1987, 31, 1388–1393. 10.1128/aac.31.9.1388. 2823697 PMC174948

[ref12] Kilmarx P. H.; Blanchard K.; Chaikummao S.; Friedland B. A.; Srivirojana N.; Connolly C.; Witwatwongwana P.; Supawitkul S.; Mock P. A.; Chaowanachan T.; et al. A Randomized, Placebo-Controlled Trial to Assess the Safety and Acceptability of Use of Carraguard Vaginal Gel by Heterosexual Couples in Thailand. Sex. Transm. Dis. 2008, 35, 226–232. 10.1097/olq.0b013e31815d6e0d. 18490865

[ref13] Rodríguez A.; Kleinbeck K.; Mizenina O.; Kizima L.; Levendosky K.; Jean-Pierre N.; Villegas G.; Ford B. E.; Cooney M. L.; Teleshova N.; et al. In Vitro and in Vivo Evaluation of Two Carrageenan-Based Formulations to Prevent HPV Acquisition. Antiviral Res. 2014, 108, 88–93. 10.1016/j.antiviral.2014.05.018. 24909570 PMC4116815

[ref14] Grassauer A.; Weinmuellner R.; Meier C.; Pretsch A.; Prieschl-Grassauer E.; Unger H. Iota-Carrageenan Is a Potent Inhibitor of Rhinovirus Infection. Virol. J. 2008, 5, 107 10.1186/1743-422x-5-107. 18817582 PMC2562995

[ref15] Koenighofer M.; Lion T.; Bodenteich A.; Prieschl-Grassauer E.; Grassauer A.; Unger H.; Mueller C. A.; Fazekas T. Carrageenan Nasal Spray in Virus Confirmed Common Cold: Individual Patient Data Analysis of Two Randomized Controlled Trials. Multidiscip. Respir. Med. 2014, 9, 57 10.1186/2049-6958-9-57. 25411637 PMC4236476

[ref16] Pereira L.; Critchley A. T. The COVID 19 Novel Coronavirus Pandemic 2020: Seaweeds to the Rescue? Why Does Substantial, Supporting Research about the Antiviral Properties of Seaweed Polysaccharides Seem to Go Unrecognized by the Pharmaceutical Community in These Desperate Times?. J. Appl. Phycol. 2020, 32, 1875–1877. 10.1007/s10811-020-02143-y. PMC7263178 32836796

[ref17] Handbook of Hydrocolloids; Phillips G. O., Williams P. A., Eds.; Woodhead Publishing Limited, 2009.

[ref18] Almutairi F. M.; Adams G. G.; Kök M. S.; Lawson C. J.; Gahler R.; Wood S.; Foster T. J.; Rowe A. J.; Harding S. E. An Analytical Ultracentrifugation Based Study on the Conformation of Lambda Carrageenan in Aqueous Solution. Carbohydr. Polym. 2013, 97, 203–209. 10.1016/j.carbpol.2013.04.027. 23769538

[ref19] An Introduction to Polysaccharide Biotechnology; Harding S. E., Tombs M. P., Adams G. G., Paulsen B. S., Inngjerdingen K. T., Barsett H., Eds.; CRC Press: Boca Raton, 2017.

[ref20] Bonferoni M. C.; Rossi S.; Tamayo M.; Pedraz J. L.; Dominguez-Gil A.; Caramella C. On the Employment of λ-Carrageenan in a Matrix System I. Sensitivity to Dissolution Medium and Comparison with Na Carboxymethylcellulose and Xanthan Gum. J. Controlled Release 1993, 26, 119–127. 10.1016/0168-3659(93)90111-h.

[ref21] Bonferoni M. C.; Rossi S.; Tamayo M.; Pedraz J. L.; Dominguez-Gil A.; Caramella C. On the Employment of λ-Carrageenan in a Matrix System II. λ-Carrageenan and Hydroxypropylmethylcellulose Mixtures. J. Controlled Release 1994, 30, 175–182. 10.1016/0168-3659(94)90264-x.

[ref22] Zhou G.; Sun Y.; Xin H.; Zhang Y.; Li Z.; Xu Z. In Vivo Antitumor and Immunomodulation Activities of Different Molecular Weight Lambda-Carrageenans from Chondrus Ocellatus. Pharmacol. Res. 2004, 50, 47–53. 10.1016/j.phrs.2003.12.002. 15082028

[ref23] Nakashima H.; Kido Y.; Kobayashi N.; Motoki Y.; Neushul M.; Yamamoto N. Purification and Characterization of an Avian Myeloblastosis and Human Immunodeficiency Virus Reverse Transcriptase Inhibitor , Sulfated Polysaccharides Extracted from Sea Algae. Antimicrob. Agents Chemother. 1987, 31, 1524–1528. 10.1128/aac.31.10.1524. 2449120 PMC174983

[ref24] Nakata R.; Miyazaki T.; Morita Y.; Ishida E.; Iwatsuki R.; Ohtsuki C. Apatite Formation Abilities of Various Carrageenan Gels in Simulated Body Environment. J. Ceram. Soc. Jpn. 2010, 118, 487–490. 10.2109/jcersj2.118.487.

[ref25] Briones A. V.; Sato T.; Bigol U. G. Antibacterial Activity of Polyethylenimine/Carrageenan Multilayer against Pathogenic Bacteria. Adv. Chem. Eng. Sci. 2014, 04, 233–241. 10.4236/aces.2014.42026.

[ref26] Sokolova E. V.; Chusovitin E. A.; Barabanova A. O.; Balagan S. A.; Galkin N. G.; Yermak I. M. Atomic Force Microscopy Imaging of Carrageenans from Red Algae of Gigartinaceae and Tichocarpaceae Families. Carbohydr. Polym. 2013, 93, 458–465. 10.1016/j.carbpol.2012.12.026. 23499083

[ref27] Schoeler B.; Delorme N.; Doench I.; Sukhorukov G. B.; Fery A.; Glinel K. Polyelectrolyte Films Based on Polysaccharides of Different Conformations: Effects on Multilayer Structure and Mechanical Properties. Biomacromolecules 2006, 7, 2065–2071. 10.1021/bm060378a. 16768435

[ref28] Briones A. V.; Sato T. 2011 International Conference on Biology, Environment and Chemistry IPCBEE. 2011 International Proceedings of Chemical, Biological and Environmental Engineering: Singapore, 2011; Vol. 24; pp 288–291.

[ref29] Elizarova I. S.; Luckham P. F. Fabrication of Polyelectrolyte Multilayered Nano-Capsules Using a Continuous Layer-by-Layer Approach. J. Colloid Interface Sci. 2016, 470, 92–99. 10.1016/j.jcis.2016.02.052. 26939072

[ref30] Oliveira S. M.; Silva T. H.; Reis R. L.; Mano J. F. Nanocoatings Containing Sulfated Polysaccharides Prepared by Layer-by-Layer Assembly as Models to Study Cell-Material Interactions. J. Mater. Chem. B 2013, 1, 4406–4418. 10.1039/c3tb20624f. 32261113

[ref31] Laufer G.; Kirkland C.; Cain A. A.; Grunlan J. C. Oxygen Barrier of Multilayer Thin Films Comprised of Polysaccharides and Clay. Carbohydr. Polym. 2013, 95, 299–302. 10.1016/j.carbpol.2013.02.048. 23618273

[ref32] Oliveira S. M.; Santo V. E.; Gomes M. E.; Reis R. L.; Mano J. F. Layer-by-Layer Assembled Cell Instructive Nanocoatings Containing Platelet Lysate. Biomaterials 2015, 48, 56–65. 10.1016/j.biomaterials.2015.01.020. 25701032

[ref33] Dul M.; Paluch K. J.; Kelly H.; Healy A. M.; Sasse A.; Tajber L. Self-Assembled Carrageenan/Protamine Polyelectrolyte Nanoplexes-Investigation of Critical Parameters Governing Their Formation and Characteristics. Carbohydr. Polym. 2015, 123, 339–349. 10.1016/j.carbpol.2015.01.066. 25843867

[ref34] Stechemesser S.; Eimer W. Solvent-Dependent Swelling of Poly(Amido Amine) Starburst Dendrimers. Macromolecules 1997, 30, 2204–2206. 10.1021/ma9614914.

[ref35] Michna A.; Pomorska A.; Nattich-Rak M.; Wasilewska M.; Adamczyk Z. Hydrodynamic Solvation of Poly(Amido Amine) Dendrimer Monolayers on Silica. J. Phys. Chem. C 2020, 124, 17684–17695. 10.1021/acs.jpcc.0c04638.

[ref36] Einstein A. Elementare Theorie der Brownschen) Bewegung. Zeitschrift für Elektrochemie und Angew. Phys. Chemie 1908, 14, 235–239. 10.1002/bbpc.19080141703.

[ref37] Electrical Phenomena at Interfaces and Biointerfaces: Fundamentals and Applications in Nano-, Bio-, and Environmental Sciences; Ohshima H., Ed.; John Wiley & Sons, Inc.: Hoboken, New Jersey, 2012.

[ref38] von Smoluchowski M. Contribution to the Theory of Electro- Osmosis and Related Phenomena. Bull. Int. Acad. Sci. Cracovie 1903, 3, 182–199.

[ref39] Morga M.; Michna A.; Adamczyk Z. Formation and Stability of Polyelectrolyte/Polypeptide Monolayers Determined by Electrokinetic Measurements. Colloids Surf., A 2017, 529, 302–310. 10.1016/j.colsurfa.2017.05.033.

[ref40] Michna A.; Adamczyk Z.; Sofińska K.; Matusik K. Monolayers of Poly(Amido Amine) Dendrimers on Mica – In Situ Streaming Potential Measurements. J. Colloid Interface Sci. 2017, 485, 232–241. 10.1016/j.jcis.2016.09.007. 27665076

[ref41] Michna A.; Batys P.; Morga M.; Pomorska A.; Wytrwal-Sarna M.; Kepczynski M.; Adamczyk Z. Formation of Strong Polycation (Poly[(3-Allylamino-2-Hydroxypropyl)Trimethylammonium Chloride]) Monolayers on Mica, Silica, and Gold Substrates: Modeling and Experimental Studies. J. Phys. Chem. C 2019, 123, 19022–19032. 10.1021/acs.jpcc.9b04533.

[ref42] Kozma P.; Hámori A.; Kurunczi S.; Cottier K.; Horvath R. Grating Coupled Optical Waveguide Interferometer for Label-Free Biosensing. Sens. Actuators, B 2011, 155, 446–450. 10.1016/j.snb.2010.12.045.

[ref43] Patko D.; Cottier K.; Hamori A.; Horvath R. Single Beam Grating Coupled Interferometry: High Resolution Miniaturized Label-Free Sensor for Plate Based Parallel Screening. Opt. Express 2012, 20, 23162–23173. 10.1364/oe.20.023162. 23188281

[ref44] De Feijter J. A.; Benjamins J.; Veer F. A. Ellipsometry as a Tool to Study the Adsorption Behavior of Synthetic and Biopolymers at the Air–Water Interface. Biopolymers 1978, 17, 1759–1772. 10.1002/bip.1978.360170711.

[ref45] Pomorska A.; Adamczyk Z.; Nattich-Rak M.; Sadowska M. Kinetics of Human Serum Albumin Adsorption at Silica Sensor: Unveiling Dynamic Hydration Function. Colloids Surf., B 2018, 167, 377–384. 10.1016/j.colsurfb.2018.04.017. 29705664

[ref46] Thành T. T. T.; Yuguchi Y.; Mimura M.; Yasunaga H.; Takano R.; Urakawa H.; Kajiwara K. Molecular Characteristics and Gelling Properties of the Carrageenan Family, 1: Preparation of Novel Carrageenans and Their Dilute Solution Properties. Macromol. Chem. Phys. 2002, 203, 15–23. 10.1002/1521-3935(20020101)203:1<15::aid-macp15>3.0.co;2-1.

[ref47] Notley S. M.; Leong Y.-K. Interaction between Silica in the Presence of Adsorbed Poly(Ethyleneimine): Correlation between Colloidal Probe Adhesion Measurements and Yield Stress. Phys. Chem. Chem. Phys. 2010, 12, 10594–10601. 10.1039/c003973j. 20601999

[ref48] Park I. H.; Choi E.-J. Characterization of Branched Polyethyleneimine by Laser Light Scattering and Viscometry. Polymers 1996, 37, 313–319. 10.1016/0032-3861(96)81104-9.

[ref49] Adamczyk Z.; Michna A.; Szaraniec M.; Bratek A.; Barbasz J. Characterization of Poly(Ethylene Imine) Layers on Mica by the Streaming Potential and Particle Deposition Methods. J. Colloid Interface Sci. 2007, 313, 86–96. 10.1016/j.jcis.2007.04.005. 17521663

[ref50] Jachimska B.; Łapczyńska M.; Zapotoczny S. Reversible Swelling Process of Sixth-Generation Poly(Amido Amine) Dendrimers Molecule as Determined by Quartz Crystal Microbalance Technique. J. Phys. Chem. C 2013, 117, 1136–1145. 10.1021/jp307832p.

[ref51] Perico A. Electrostatic Theory of the Assembly of PAMAM Dendrimers and DNA. Biopolymers 2016, 105, 276–286. 10.1002/bip.22805. 26756793

[ref52] Prosa T. J.; Bauer B. J.; Amis E. J. From Stars to Spheres: A SAXS Analysis of Dilute Dendrimer Solutions. Macromolecules 2001, 34, 4897–4906. 10.1021/ma0002186.

[ref53] Nisato G.; Ivkov R.; Amis E. J. Size Invariance of Polyelectrolyte Dendrimers. Macromolecules 2000, 33, 4172–4176. 10.1021/ma991474p.

[ref54] Jackson C. L.; Chanzy H. D.; Booy F. P.; Drake B. J.; Tomalia D. A.; Bauer B. J.; Amis E. J. Visualization of Dendrimer Molecules by Transmission Electron Microscopy (TEM): Staining Methods and Cryo-TEM of Vitrified Solutions. Macromolecules 1998, 31, 6259–6265. 10.1021/ma9806155.

[ref55] Pfau A.; Schrepp W.; Horn D. Detection of a Single Molecule Adsorption Structure of Poly(Ethylenimine) Macromolecules by AFM†. Langmuir 1999, 15, 3219–3225. 10.1021/la9808925.

[ref56] Schneider M.; Zhu M.; Papastavrou G.; Akari S.; Möhwald H. Chemical Pulsed-Force Microscopy of Single Polyethyleneimine Molecules in Aqueous Solution. Langmuir 2002, 18, 602–606. 10.1021/la0113116.

[ref57] Saftics A.; Agócs E.; Fodor B.; Patkó D.; Petrik P.; Kolari K.; Aalto T.; Fürjes P.; Horvath R.; Kurunczi S. Investigation of Thin Polymer Layers for Biosensor Applications. Appl. Surf. Sci. 2013, 281, 66–72. 10.1016/j.apsusc.2012.12.042.

[ref58] Longtin R.; Maroni P.; Borkovec M. Transition from Completely Reversible to Irreversible Adsorption of Poly(Amido Amine) Dendrimers on Silica. Langmuir 2009, 25, 2928–2934. 10.1021/la8038818. 19437704

[ref59] Meakin P.; Jullien R. Random-Sequential Adsorption of Disks of Different Sizes. Phys. Rev. A: At., Mol., Opt. Phys. 1992, 46, 2029–2038. 10.1103/physreva.46.2029. 9908338

[ref60] Adamczyk Z.; Siwek B.; Zembala M.; Weroński P. Influence of Polydispersity on Random Sequential Adsorption of Spherical Particles. J. Colloid Interface Sci. 1997, 185, 236–244. 10.1006/jcis.1996.4540. 9056341

[ref61] Oćwieja M.; Matras-Postołek K.; Maciejewska-Prończuk J.; Morga M.; Adamczyk Z.; Sovinska S.; Żaba A.; Gajewska M.; Król T.; Cupiał K.; et al. Formation and Stability of Manganese-Doped ZnS Quantum Dot Monolayers Determined by QCM-D and Streaming Potential Measurements. J. Colloid Interface Sci. 2017, 503, 186–197. 10.1016/j.jcis.2017.04.059. 28525826

[ref62] Adamczyk Z. Particles at Interfaces; Academic Press, 2017.

[ref63] Morga M.; Adamczyk Z. Monolayers of Cationic Polyelectrolytes on Mica - Electrokinetic Studies. J. Colloid Interface Sci. 2013, 407, 196–204. 10.1016/j.jcis.2013.05.069. 23849822

[ref64] Mészáros R.; Thompson L.; Bos M.; De Groot P. Adsorption and Electrokinetic Properties of Polyethylenimine on Silica Surfaces. Langmuir 2002, 18, 6164–6169. 10.1021/la011776w.

[ref65] Cahill B. P.; Papastavrou G.; Koper G. J. M.; Borkovec M. Adsorption of Poly(Amido Amine) (PAMAM) Dendrimers on Silica: Importance of Electrostatic Three-Body Attraction. Langmuir 2008, 24, 465–473. 10.1021/la7021352. 18072793

[ref66] Porus M.; Clerc F.; Maroni P.; Borkovec M. Ion-Specific Responsiveness of Polyamidoamine (PAMAM) Dendrimers Adsorbed on Silica Substrates. Macromolecules 2012, 45, 3919–3927. 10.1021/ma3004295.

[ref67] Mészáros R.; Varga I.; Gilányi T. Adsorption of Poly(Ethyleneimine) on Silica Surfaces: Effect of PH on the Reversibility of Adsorption. Langmuir 2004, 20, 5026–5029. 10.1021/la049611l. 15984264

[ref68] Adamczyk Z.; Sadowska M. Hydrodynamic Solvent Coupling Effects in Quartz Crystal Microbalance Measurements of Nanoparticle Deposition Kinetics. Anal. Chem. 2020, 92, 3896–3903. 10.1021/acs.analchem.9b05397. 31994383 PMC7588021

[ref69] Åkesson A.; Lundgaard C. V.; Ehrlich N.; Pomorski T. G.; Stamou D.; Cárdenas M. Induced Dye Leakage by PAMAM G6 Does Not Imply Dendrimer Entry into Vesicle Lumen. Soft Matter 2012, 8, 8972–8980. 10.1039/c2sm25864a.

[ref70] Johannsmann D. Viscoelastic, Mechanical, and Dielectric Measurements on Complex Samples with the Quartz Crystal Microbalance. Phys. Chem. Chem. Phys. 2008, 10, 4516–4534. 10.1039/b803960g. 18665301

[ref71] Reviakine I.; Johannsmann D.; Richter R. P. Hearing What You Cannot See and Visualizing What You Hear: Interpreting Quartz Crystal Microbalance Data from Solvated Interfaces. Anal. Chem. 2011, 83, 8838–8848. 10.1021/ac201778h. 21939220

[ref72] Sauerbrey G. n. Verwendung von Schwingquarzen Zur Wägung Dünner Schichten Und Zur Mikrowägung. Z. Phys. 1959, 155, 206–222. 10.1007/bf01337937.

[ref73] Elzbieciak M.; Zapotoczny S.; Nowak P.; Krastev R.; Nowakowska M.; Warszyński P. Influence of PH on the Structure of Multilayer Films Composed of Strong and Weak Polyelectrolytes. Langmuir 2009, 25, 3255–3259. 10.1021/la803988k. 19437787

[ref74] Adamczyk Z.; Morga M.; Kosior D.; Batys P. Conformations of Poly-L-Lysine Molecules in Electrolyte Solutions: Modeling and Experimental Measurements. J. Phys. Chem. C 2018, 122, 23180–23190. 10.1021/acs.jpcc.8b07606.

[ref75] Kosior D.; Morga M.; Maroni P.; Cieśla M.; Adamczyk Z. Formation of Poly - L - Lysine Monolayers on Silica: Modeling and Experimental Studies. J. Phys. Chem. C 2020, 124, 4571–4581. 10.1021/acs.jpcc.9b10870.

[ref76] Tokarczyk K.; Jachimska B. Quantitative Interpretation of PAMAM Dendrimers Adsorption on Silica Surface. J. Colloid Interface Sci. 2017, 503, 86–94. 10.1016/j.jcis.2017.05.002. 28500943

[ref77] Mureşan L.; Maroni P.; Popa I.; Porus M.; Longtin R.; Papastavrou G.; Borkovec M. Conformational Changes of Polyamidoamine (PAMAM) Dendrimers Adsorbed on Silica Substrates. Macromolecules 2011, 44, 5069–5071. 10.1021/ma201103n.

[ref78] Karabulut E.; Wågberg L. Design and Characterization of Cellulose Nanofibril-Based Freestanding Films Prepared by Layer-by-Layer Deposition Technique. Soft Matter 2011, 7, 3467–3474. 10.1039/c0sm01355b.

