# Supplementary material for: Effect of the Anchoring Layer and Transport Type on the Adsorption Kinetics of Lambda Carrageenan
Source: J Phys Chem B. 2021 Jul 13;125(28):7797–808. doi: 10.1021/acs.jpcb.1c03550 (PMC8389906; doi:10.1021/acs.jpcb.1c03550)
Supplement: Supplementary file 1 — jp1c03550_si_001.pdf [file jp1c03550_si_001.pdf]

Supporting Information for Publication

Effect of the Anchoring Layer and Transport Type on the Adsorption Kinetics of Lambda Carrageenan

Aneta Michna*[1], Julia Maciejewska-Prończuk[1], Agata Pomorska[1], Monika Wasilewska[1], Tayfun Kilicer[2], Julia Witt[2], Ozlem Ozcan[2]

[1] Jerzy Haber Institute of Catalysis and Surface Chemistry, Polish Academy of Sciences, Niezapominajek 8, PL-30239 Krakow, Poland.

[2] Bundesanstalt für Materialforschung und -prüfung, Unter den Eichen 87, 12163, Berlin, Germany

E-mail addresses: aneta.michna@ikifp.edu.pl (A.Michna)
julia.maciejewska-pronczuk@ikifp.edu.pl
agata.pomorska@ikifp.edu.pl
monika.wasilewska@ikifp.edu.pl
tayfun.kilicer@campus.tu-berlin.de
julia.witt@bam.de
ozlem.ozcan@bam.de

*Corresponding author

Aneta Michna

Jerzy Haber Institute of Catalysis and Surface Chemistry, Polish Academy of Sciences, Niezapominajek 8, PL-30239 Krakow, Poland

phone: +48126395202; fax: +48124251923

e-mail: aneta.michna@ikifp.edu.pl

Contents:

1. Size and zeta potential distributions of the macroions
2. Stability of the macroion layers determined from streaming potential measurements
3. Frequency (ΔFq) and dissipation (ΔD) shift as a function of macroion adsorption time

1. Size and zeta potential distributions of the macroions

In Figure S1, the typical hydrodynamic diameter (size) distribution by Number evaluated for lambda carrageenan (λ-car) a), branched polyethyleneimine (bPEI) b), and poly(amidoamine) dendrimers (PAMAMD) c) were presented. The results were derived from the Zeta Sizer using the dynamic light scattering technique. The data were collected for the macroion bulk concentration of 100 mg L^{-1}, in 0.01 M NaCl at pH 5.8. As can be observed, the maximum peak appeared at 69.7 nm for lambda carrageenan (see Figure S1a), at 14 nm for branched polyethyleneimine (see Figure S1b), and at 10 nm for the eighth generation of poly(amidoamine) dendrimers (see Figure. S1c).

a) λ-car

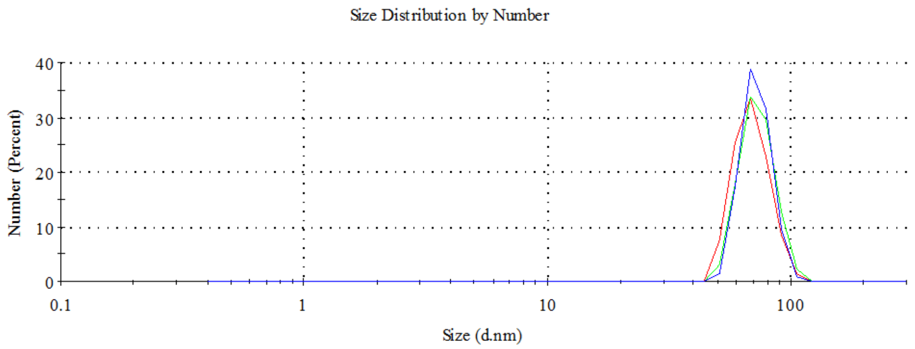

b) bPEI

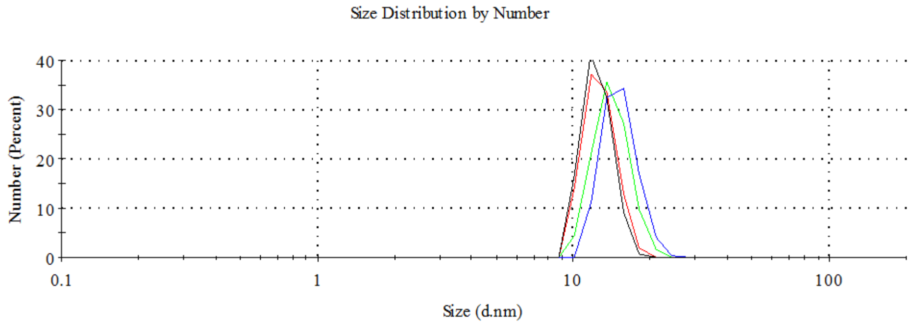

c) PAMAMD

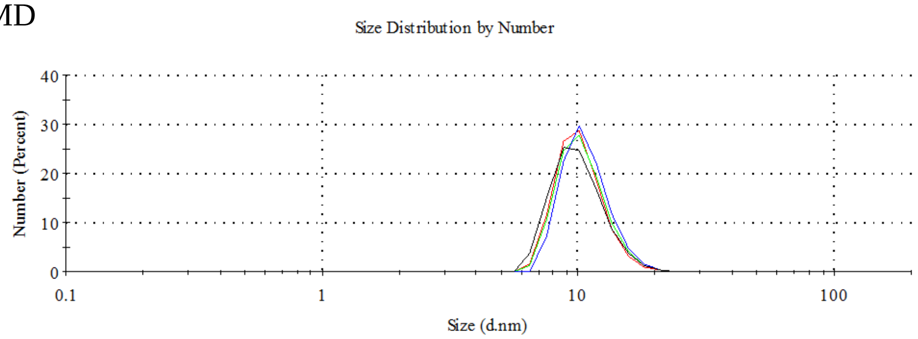

Figure S1 Size distributions by Number of a) λ-car; b) bPEI c) PAMAMD determined for the macroion concentration of 100 mg L^{-1} in the ionic strength of 0.01 M NaCl at pH 5.8. The measurements were performed using the dynamic light scattering method.

As shown in Figure S2 a) the strong maximum peak at -86 mV appeared in the zeta potential distribution of lambda carrageenan, which indicates that this macroanion is strongly negatively charged. Contrary to carrageenan, the branched polyethyleneimine (see Figure S2 b), and poly(amidoamine) dendrimers (Figure S2 c) are strongly positively charged. The maximum peaks appeared at 70 mV and 65 mV (see Figure S2 b and c) for bPEI and PAMAMD, respectively).

a) λ-car

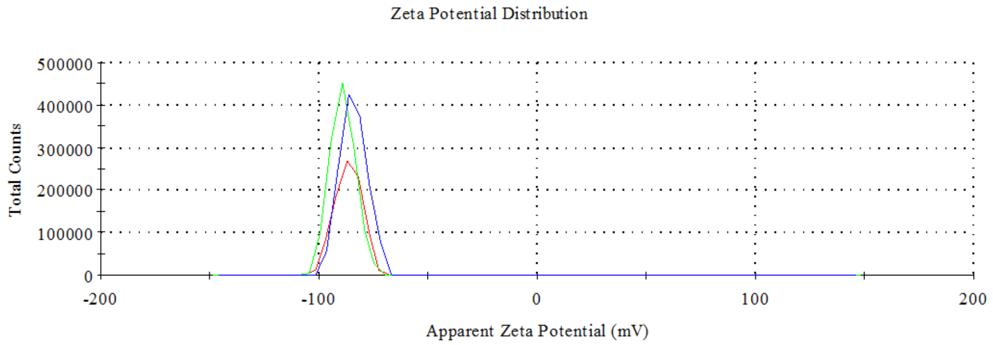

b) bPEI

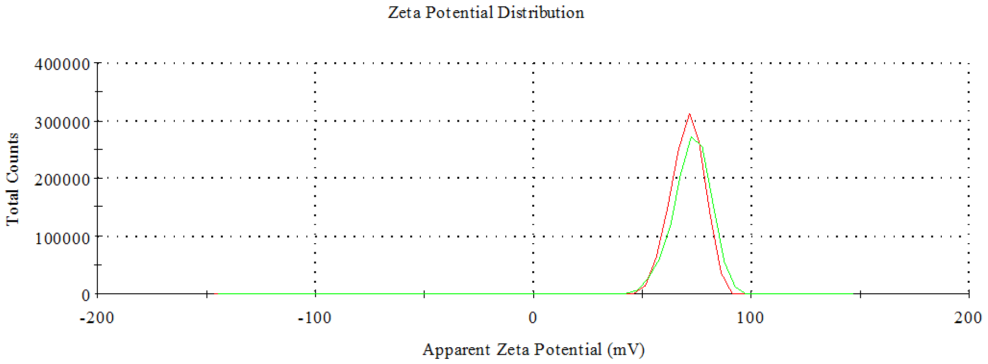

c) PAMAMD

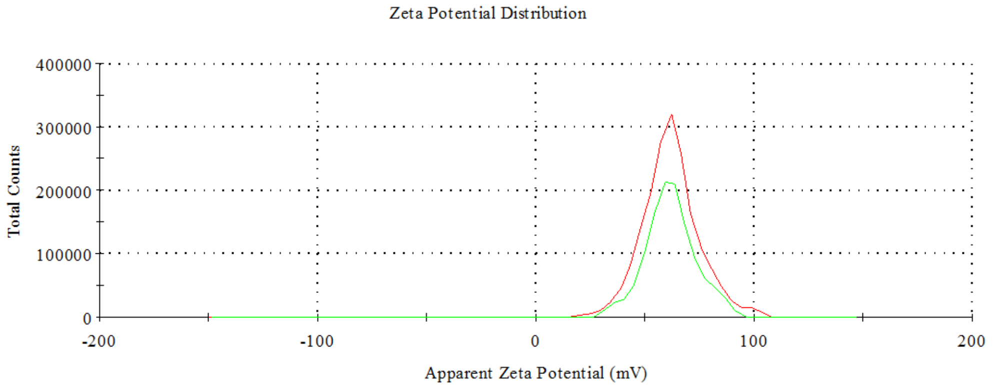

Figure S2 Zeta potential distribution of a) λ-car; b) bPEI c) PAMAMD determined for the macroion concentration of 500 mg L^{-1} in the ionic strength of 0.01 M NaCl at pH 5.8. The data were determined from electrophoretic measurements using electrophoresis combined with laser Doppler velocimetry technique.

2. Stability of the macroion layers determined from streaming potential measurements

The examples of the desorption of the macroions from either pure or macroion modified SiO_2 wafers, obtained from the streaming potential measurements, were presented in Figure S3 as the dependence of the mono- or bilayer zeta potential on the rinsing time (ionic strength 0.01 M, pH 5.8).

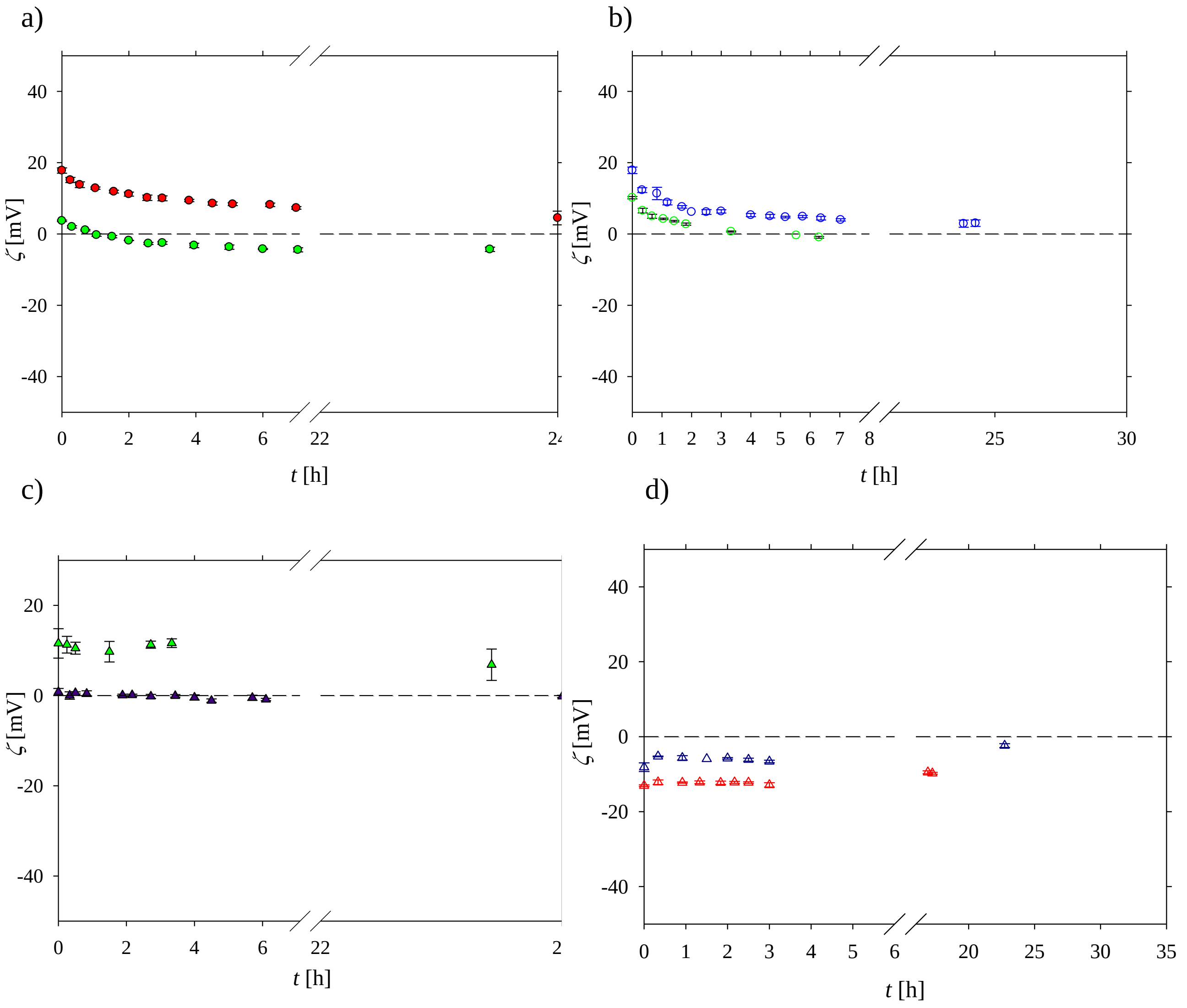

Figure S3 The dependencies of the apparent zeta potential of a) PAMAMD (●,●), b) bPEI (○,○), c) PAMAMD/ λ-car (▲,▲), d) bPEI/ λ-car (Δ, Δ) layer covered SiO_2 wafer on the desorption time *t*. The pure electrolyte flow rate 2.0×10^{-2} cm^3 s^{-1}, ionic strength I=0.01 M NaCl, pH 5.8. The macroions were adsorbed under diffusion controlled transport conditions.

The initial bulk concentrations and adsorption time of the layer/ bilayer formation were as follow: PAMAMD: 2 mg L^{-1} 1.5 min (●); PAMAMD: 2 mg L^{-1} 18 min (●); bPEI: 2 mg L^{-1} 0.5 min (○); bPEI: 2 mg L^{-1} 20 min (○); PAMAMD: 5 mg L^{-1} 10 min/ λ-car: 5 mg L^{-1} 0.5 min (▲); PAMAMD: 5 mg L^{-1} 10 min/ λ-car: 5 mg L^{-1} 16 min (▲); bPEI: 5 mg L^{-1} 10 min/ λ-car: 5 mg L^{-1} 3 min (Δ); bPEI: 5 mg L^{-1} 10 min/ λ-car: 5 mg L^{-1} 25 min (Δ).

As can be seen, the zeta potential of the monolayers formed by PAMAMD (Figure S3a) and bPEI (Figure S3b) decreased monotonically with the rinsing time for all various macroion surface concentration (expressed as various values of the apparent zeta potential). In all cases, the final zeta potential after 7 h of rinsing was smaller than the initial zeta potential indicating that small fraction of these macroions was desorbed from the pure SiO_2 wafer during this period.

The stabilities of the PAMAMD/ λ-car and bPEI/ λ-car bilayers (Figure S3c-d) were also determined. As can be observed, oppositely to the monolayers, the λ-car, forming the outer layer, did not tend to desorb from the surface.

Accordingly, the desorption experiments, performed using streaming potential measurements, confirmed that λ-car layers are stable within the experiment period reaching 7h.

3.Frequency (ΔFq) and dissipation (ΔD) shift as a function of macroion adsorption time

Figure S4 presents the formation of PAMAMD /λ-car bilayer in NaCl with ionic strength of 0.01 M at pH of 5.8 as mean frequency (ΔFq) and mean dissipation (ΔD) shifts as a function of time, monitored through QCM technique.

a)

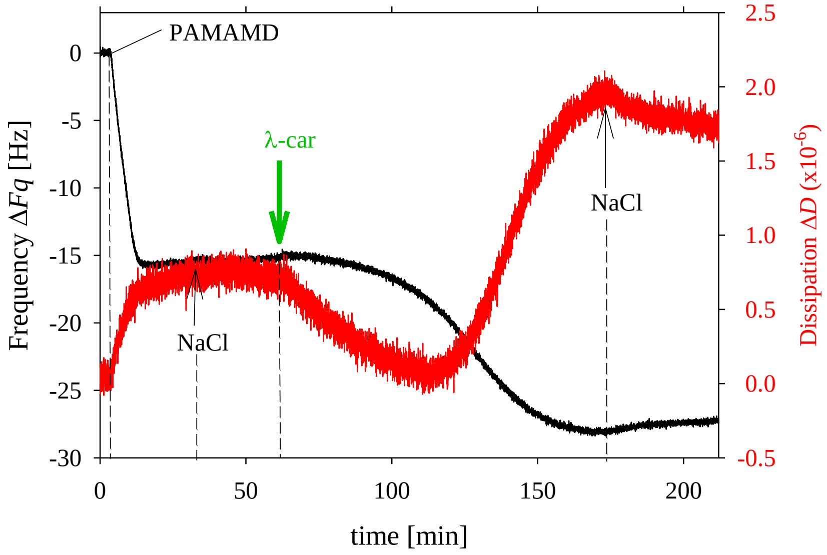

b)

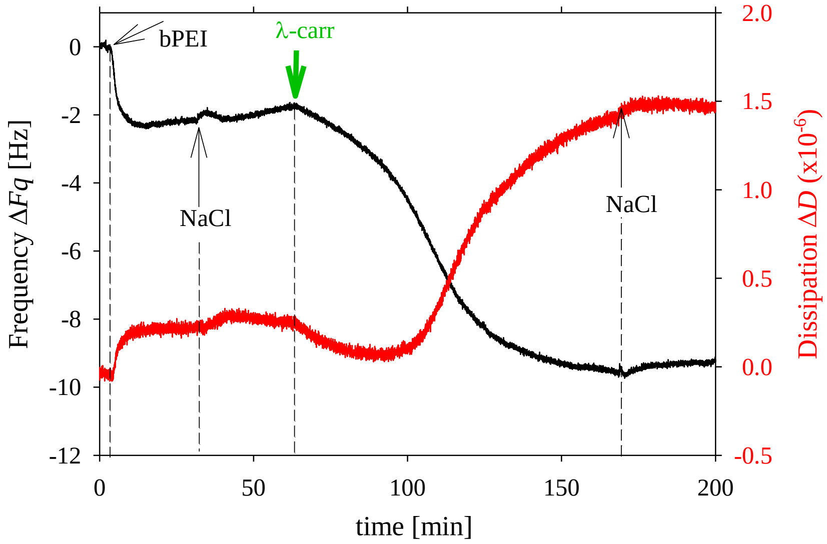

Figure S4 The dependences of mean frequency changes (ΔFq) and mean dissipation (ΔD) shift on time measured during the formation of: Part a) PAMAMD/ λ-car and Part b) bPEI /λ-car bilayers. The mass concentration of the macroions was 1 mg L^{-1}, ionic strength of 0.01 M, pH 5.8.

PAMAMD adsorption brought out the following QCM parameters shifts: ΔFq=-15Hz and ΔD=0.75 × 10^{-6}. Introduction of 1 mg L^{-1} λ-car solution (green arrow on the graph) led to non-linear decrease of ΔFq down to – 28 Hz in combination with the decrease of ΔD to zero value within the first 50 min followed by an increase up to 2.0 × 10^{-6} unit (110 min from the green arrow), when the cell was purged with electrolyte again.

A similar trend of ΔD was monitored during the deposition of λ-car on bPEI monolayer (Figure S4b) in the same conditions as the previous experiment (electrolyte: NaCl, ionic strength of 0.01 M, pH= 5.8, the concentration of bPEI and λ-car=1 mg L^{-1}). Adsorption of bPEI monolayer from 1 mg L^{-1} solution for 30 minutes led to ΔFq of -2 Hz and ΔD of 0.2 × 10^{-6} unit. The subsequent introduction of 1 mg L^{-1} solution of λ-car to the system (green arrow on the graph- Figure S4b) brought out far more interesting QCM output. 100 minutes of 2nd layer adsorption results with ΔFq=-9.5 Hz and ΔD=1.3 × 10^{-6}. The second parameter ΔD, related to the viscoelastic movement of the adsorbate at the quartz/ solution interface, decreases after purging the QCM cell with λ-car solution by 0.2 × 10^{-6} unit for the first 20min of the second layer deposition. Afterwards, the parameter increases up to a final value of 1.3 × 10^{-6}, when the system was purged with electrolyte.

Figure S5 presents the formation of PAMAMD /λ-car bilayer in NaCl with ionic strength of 0.01 M at pH of 5.8 as frequency (ΔFq) and dissipation (ΔD) shifts on overtones (3rd-11th) as a function of time, monitored through QCM technique.

a)

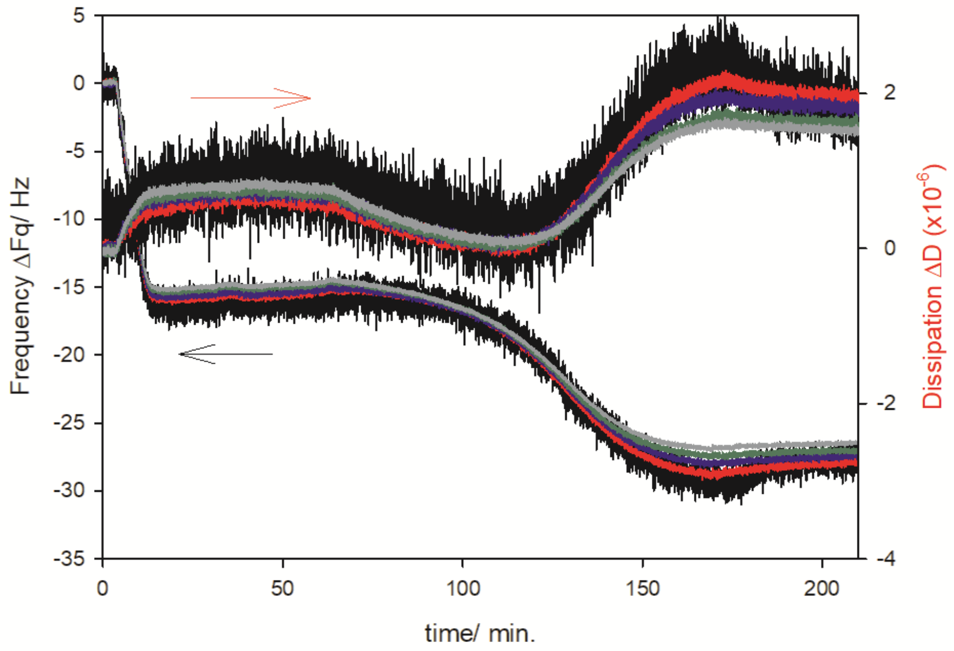

b)

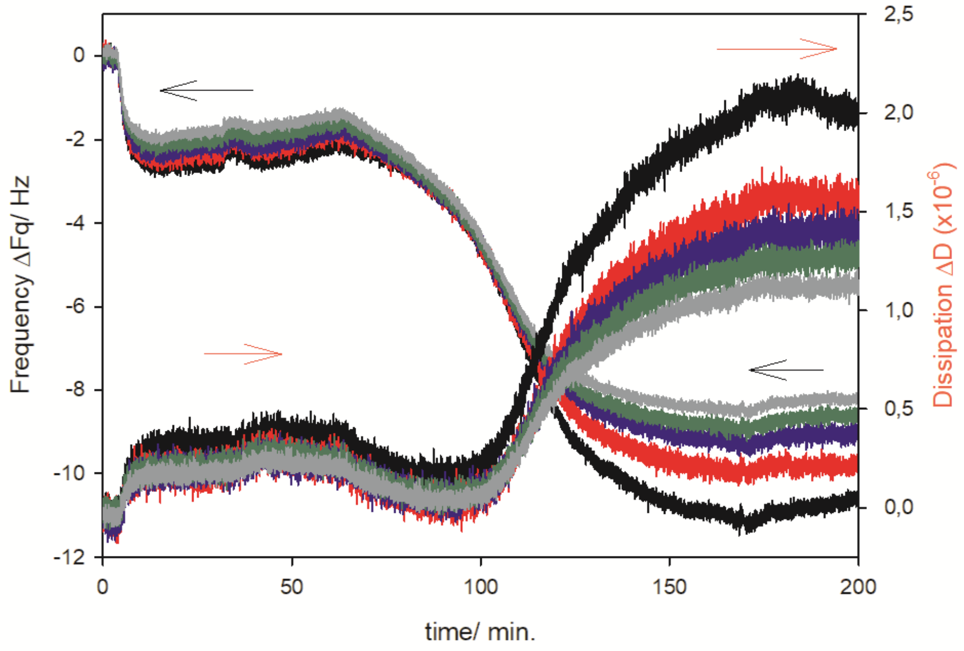

Figure S5 The dependences of frequency changes (ΔFq) and dissipation (ΔD) shift on overtones (3rd-11th) measured during the formation of: Part a) PAMAMD/ λ-car and Part b) bPEI /λ-car bilayers. The mass concentration of the macroions was 1 mg L^{-1}, ionic strength of 0.01 M, pH 5.8.
